# Complete mitogenome assembly of *Selenicereus monacanthus* revealed its molecular features, genome evolution, and phylogenetic implications

**DOI:** 10.1186/s12870-023-04529-9

**Published:** 2023-11-04

**Authors:** Guilong Lu, Wenhua Wang, Juan Mao, Qing Li, Youxiong Que

**Affiliations:** 1 College of Horticulture and Landscape Architecture, Henan Institute of Science and Technology, Xinxiang, 453003 China; 2 Institute of Vegetables, Tibet Academy of Agricultural and Animal Husbandry Sciences, Lhasa, 890032 China; 3 College of Agriculture, Fujian Agriculture and Forestry University, Fuzhou, 350002 China

**Keywords:** *Selenicereus Monacanthus*, Mitogenome, RNA editing, Gene loss, Evolution analysis

## Abstract

**Background:**

Mitochondria are the powerhouse of the cell and are critical for plant growth and development. Pitaya (*Selenicereus* or *Hylocereus*) is the most important economic crop in the family Cactaceae and is grown worldwide, however its mitogenome is unreported.

**Results:**

This study assembled the complete mitogenome of the red skin and flesh of pitaya (*Selenicereus monacanthus*). It is a full-length, 2,290,019 bp circular molecule encoding 59 unique genes that only occupy 2.17% of the entire length. In addition, 4,459 pairs of dispersed repeats (≥ 50 bp) were identified, accounting for 84.78% of the total length, and three repeats (394,588, 124,827, and 13,437 bp) mediating genomic recombination were identified by long read mapping and Sanger sequencing. RNA editing events were identified in all 32 protein-coding genes (PCGs), among which four sites (*nad1*-2, *nad4L*-2, *atp9*-copy3-223, and *ccmFC*-1309) were associated with the initiation or termination of PCGs. Seventy-eight homologous fragments of the chloroplast genome were identified in the mitogenome, the longest having 4,523 bp. In addition, evolutionary analyses suggest that *S. monacanthus* may have undergone multiple genomic reorganization events during evolution, with the loss of at least nine PCGs (*rpl2*, *rpl10*, *rps2*, *rps3*, *rps10*, *rps11*, *rps14*, *rps19*, and *sdh3*).

**Conclusions:**

This study revealed the genetic basis of the *S. monacanthus* mitogenome, and provided a scientific basis for further research on phenotypic traits and germplasm resource development.

**Supplementary Information:**

The online version contains supplementary material available at 10.1186/s12870-023-04529-9.

## Introduction

Mitochondria are important organelles within eukaryotic cells that are central to cellular respiration and energy metabolism [1]. They originated from endosymbiotic Alphaproteobacteria and became semi-autonomous organelles by gradually reducing their autonomy through gene transfer to the host cell nucleus [2, 3]. Mitochondria are maternally inherited in plants [4], except in some plants such as *Chlorophytum* [5] and *Cucumis* [6], and the green alga, *Chlamydomonas* [7]. There are various types of plant mitogenomes (including circular, linear, and reticulate) [8], with genome sizes ranging from 66 Kbp (*Viscum scurruloideum*) [9] to 11.7 Mbp (*Larix sibirica* Ledeb.) [10], and large interspecies variation within the same genus [11, 12]. The sequence and structure of plant mitogenomes are highly variable owing to widespread horizontal gene transfer and genome rearrangement [13]. However, the mitogenomes of higher plants have a smaller gene density, with their gene coding regions typically accounting for approximately 10% of their mitogenome, together with many repetitive sequences [14] and RNA editing sites [15]. Simple sequence repeats (SSRs) in plant mitogenomes are often used as genetic markers [16]. Therefore, plant mitogenomes have become important tools for species identification, phylogenetic analysis, and inheritance patterns [17, 18].

Mitochondria play an important role in plant development, ecological adaptation, and reproduction [1, 19]. In the mitochondrial genome, due to the frequent insertion/loss of genes, gene fragments or non-coding sequences, and repetitive sequence recombination, the normal functional exercise of mitochondrial genes are largely affected and thus the agronomic traits in plants are altered [8, 13, 20]. Numerous studies show that plant mitochondria are closely associated with traits including stress tolerance, plant growth vigor [21, 22], and cytoplasmic male sterility [23, 24]. Dispersed repeats (also known as transposable elements) are a class of DNA sequences that can move their position on the genome, regulate gene expression, and influence plant phenotypic traits [25] such as fruit shape in tomatoes [26], fruit color in apples [27], and plant and ear height in maize [28]. However, the abundance of repetitive sequences and complex physical structures make the assembly of complete plant mitogenome sequences particularly difficult, with complete mitogenomes reported for only 602 species to date. This is much lower than the number of chloroplast (No. 10,479) and plastid (No. 1,301) genomes (April 5, 2023, https://www.ncbi.nlm.nih.gov/genome/browse/#;/organelles/). Therefore, assembling and deciphering the mitogenome of a species is important for a deeper understanding of its genetic characteristics and for breeding research.

Pitaya (also known as pitahaya or dragon fruit) belongs to the genus *Selenicereus* or *Hylocereus* of the Cactaceae family [29, 30]. It originated in Costa Rica, Mexico, Colombia, and other Central American regions and is now widely grown in tropical and subtropical regions [31]. It is nutritionally rich and unique in function; it contains plant albumin, betaine, and water-soluble dietary fibers that are rarely found in general plants, and has high ornamental and medicinal value [32, 33]. The pitaya industry has rapidly developed in recent years, and there is an urgent need for high yielding quality varieties that are resistant to the biotic and abiotic stresses associated with production [34]. Basic genetic research on pitaya is important to further promote the use of superior germplasm resources, improve agronomic traits, and ensure industrial safety [35–37]; however, studies on its mitochondrial genome are lacking.

This study chose red skin and red flesh pitaya (*Selenicereus monacanthus*) to conduct the following research: (1) assemble the mitochondrial genome and describe its features, (2) identify repetitive sequences and predict recombination, (3) predict and validate the presence of RNA editing events, (4) assemble its chloroplast genome and identify homologous fragments with the mitogenome, and (5) perform phylogenetic and synteny analysis of closely related species. We expect this study to provide a scientific and theoretical basis for an in-depth understanding of the genetic characteristics and evolutionary history of *S. monacanthus*.

## Results

### *S. monacanthus* mitogenome assembly

**Fig. 1:**
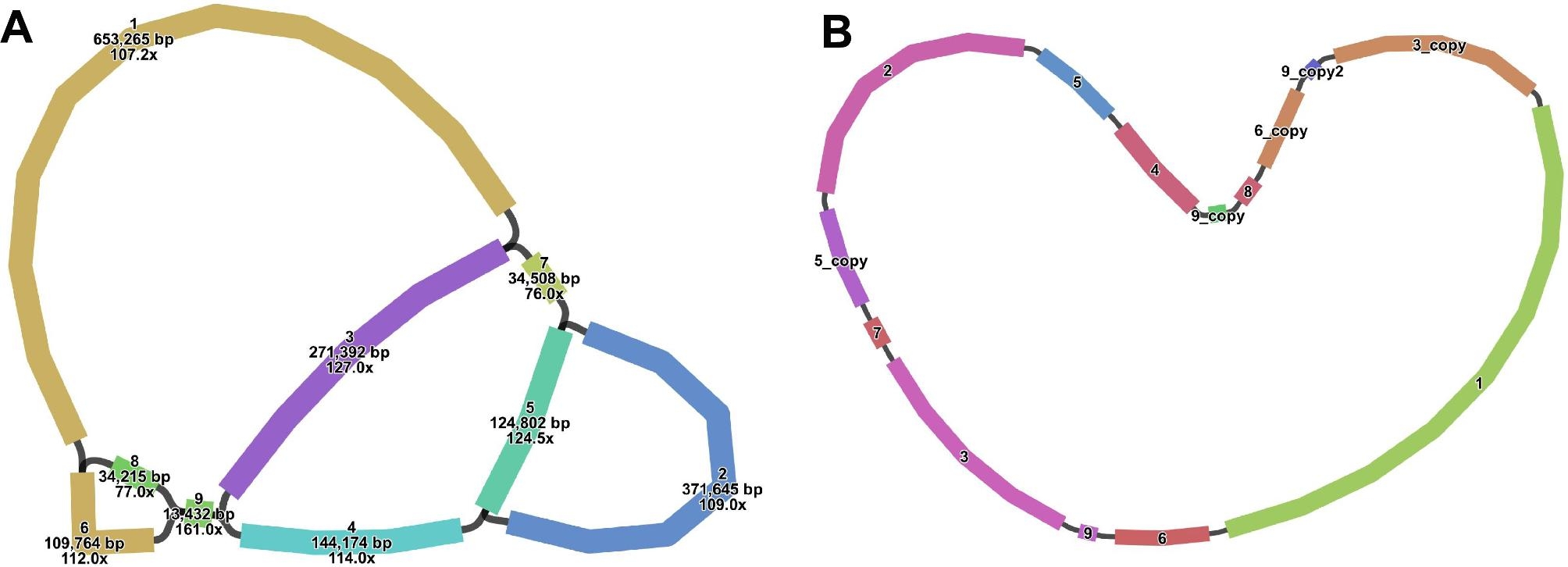
* S. monacanthus* mitogenome sketch (**A**) and master circle structure (**B**)

The *S. monacanthus* mitogenome was assembled using 10.50 Gb short-reads and 9.62 Gb long-reads using a hybrid assembly strategy. The genome sketch contains nine contigs (Fig. 1A). Contig1 and contig9 are the longest and shortest with lengths of 653,265 bp and 13,432 bp, respectively, and they also include three double bifurcating structures. We obtained a simplified main circular structure (Fig. 1B) with a total length of 2,290,019 bp after excluding the repetitive regions using the Nanopore data. This genome size was significantly larger than that of *Pereskia aculeata* (515,187 bp, NC_067638.1) of the same family [38].

### Molecular features

**Fig. 2:**
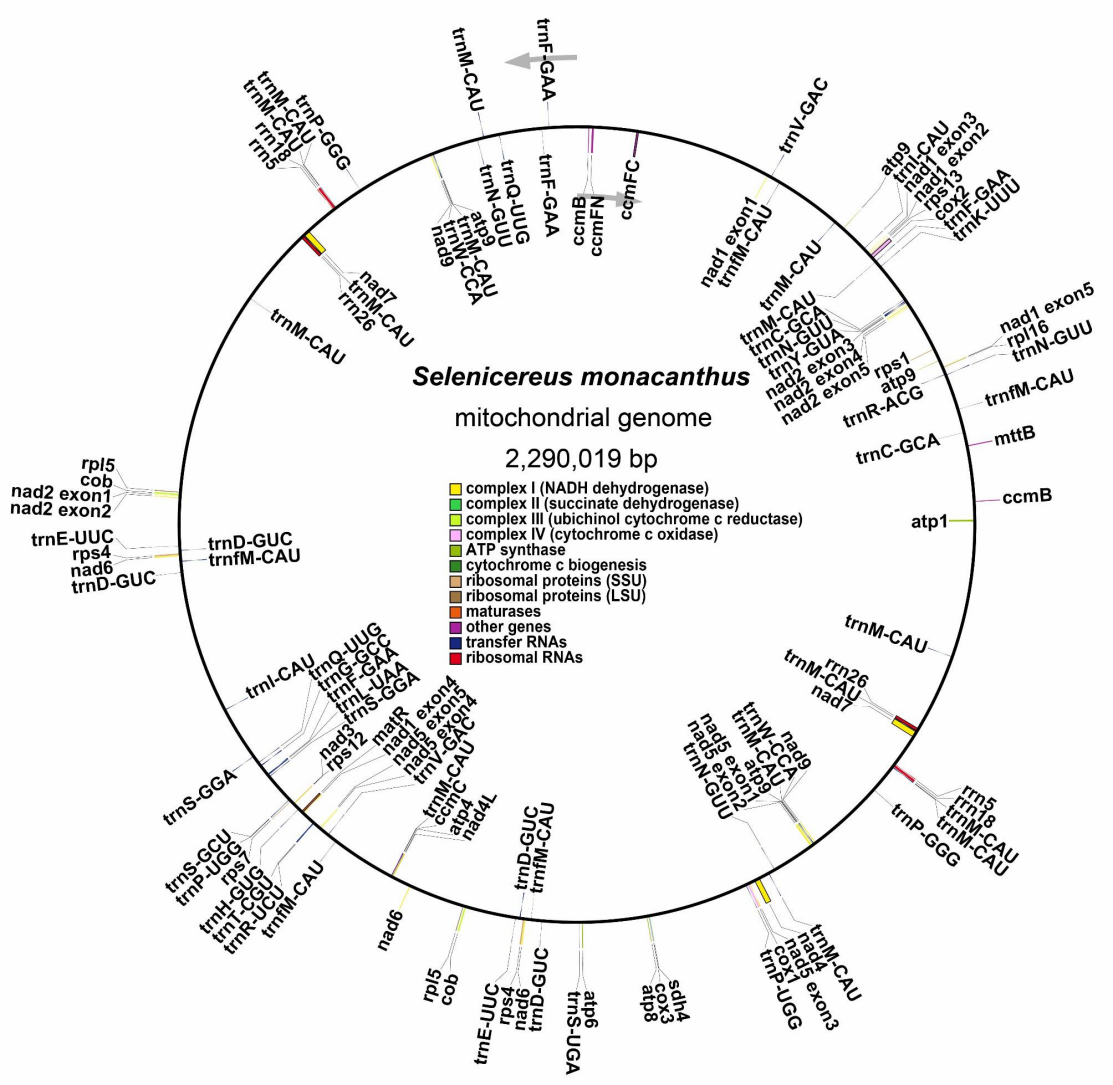
Schematic mitogenome diagram of *S. monacanthus*. Genes belonging to different functional groups are color-coded

The GC content of the *S. monacanthus* mitogenome was 43.37%, with adenine (A), thymine (T), cytosine (C), and guanine (G) representing 28.25%, 28.38%, 21.74%, and 21.63%, respectively. Thirty-two unique PCGs were annotated, including 24 core genes and eight non-core genes, as well as 24 tRNA genes (of which 14 tRNAs were multiple copies), and three rRNA genes (all multiple copies) (Fig. 2; Table 1). Nine genes were lost in the *S. monacanthus* mitogenome compared to the PCGs of “fossilized” *Liriodendron tulipifera* (*rpl2*, *rpl10*, *rps2*, *rps3*, *rps10*, *rps11*, *rps14*, *rps19*, and *sdh3*) [39], while *rps3* was present in *P. aculeata* of the same family [38]. In addition, the total length of the PCGs (35,235 bp), tRNA (4,427 bp), and rRNA (10,066 bp) coding sequences was 49,728 bp. This accounted for 2.17% of the whole genome, while over 97% of the regions were intergenic.

**Table 1:** Gene composition in the *S. monacanthus* mitogenome

Group of genes		Name of genes
Core genes	ATP synthase	*atp1, atp4, atp6, atp8, atp9* (×4)
	NADH dehydrogenase	*nad1, nad2, nad3, nad4, nad4L, nad5, nad6* (×3), *nad7* (×2), *nad9* (×2)
	Cytochrome *b*	*cob* (×2)
	Cytochrome *c* biogenesis	*ccmB* (×2), *ccmC, ccmFC, ccmFN*
	Maturases	*matR*
	Protein transport subunit	*mttB*
Variable genes	Ribosomal protein large subunit	*rpl5* (×2), *rpl16*
	Ribosomal protein small subunit	*rps1, rps4* (×2), *rps7, rps12, rps13*
	Succinate dehydrogenase	*sdh4*
rRNA genes	Ribosome RNA	*rrn5* (×2), *rrn18* (×2), *rrn26* (×2)
tRNA genes	Transfer RNA	*trnC-GCA* (×2), *trnD-GUC* (×4), *trnE-UUC* (×2), *trnF-GAA* (×4), *trnfM-CAU* (×5), *trnG-GCC, trnH-GUG, trnI-CAU* (×2), *trnK-UUU, trnL-UAA, trnM-CAU* (×14), *trnN-GUU* (×4), *trnP-GGG* (×2), *trnP-UGG* (×2), *trnQ-UUG* (×2), *trnR-ACG, trnR-UCU, trnS-GCU, trnS-GGA* (×2), *trnS-UGA, trnT-CGU, trnV-GAC* (×2), *trnW-CCA* (×2), *trnY-GUA*

Note: The number in brackets represents the copy number of the gene

The region of the *S. monacanthus* mitogenome encoding amino acids developed a unique codon usage preference during the evolution of plant adaptation (Fig. S1 and Table S1). There was a general codon preference for leucine (Leu: UUA, RSCU = 1.59), alanine (Ala: GCU, RSCU = 1.58), and a non-preference for glutamine (Gln: CAG, RSCU = 0.46) and tyrosine (Tyr: UAC, RSCU = 0.50), while the universal start codon was AUG and that for tryptophan was only UGG (both RSCUs = 1.00). The termination codon (End) preferred UAA (RSCU = 1.55), and not UAG (RSCU = 0.41).

### Repeat elements and repeat-mediated recombination

**Fig. 3:**
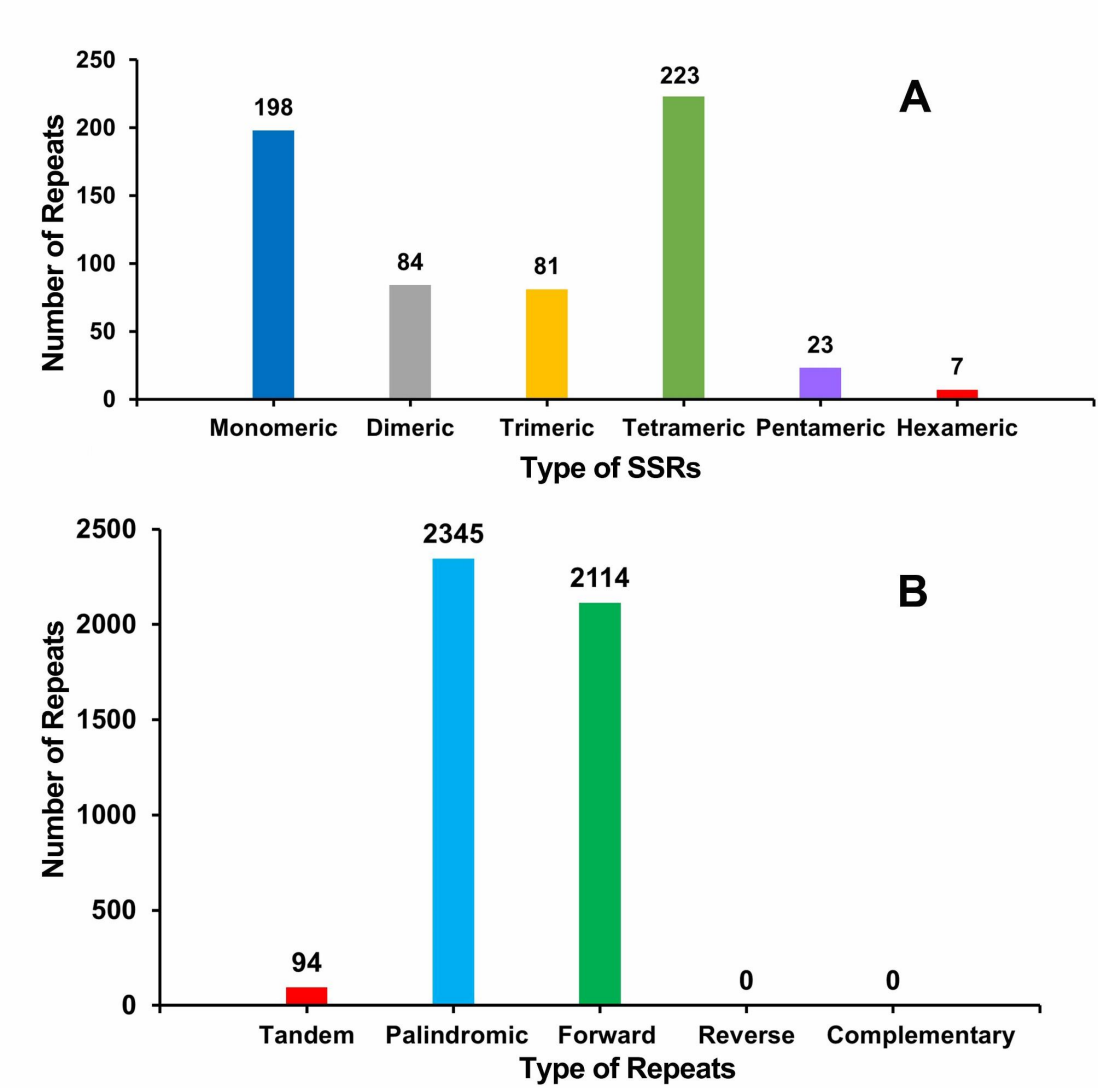
Type and number of SSRs and repeats in the *S. monacanthus* mitogenome. (**A)** Type and number of SSRs in the *S. monacanthus* mitogenome. The dark blue, gray, orange, light green, purple, and red legend indicates monomeric, dimeric, trimeric, tetrameric, pentameric, and hexameric SSRs, respectively. (**B)** Type and number of repeats in the *S. monacanthus* mitogenome. The red, blue, and green legend indicates tandem repeats, palindromic repeats, and forward repeats, respectively

In the *S. monacanthus* mitogenome, several repetitive sequences were observed (Fig. S2). A total of 616 SSRs were identified (Fig. 3A and Table S2), with the monomeric and dimeric forms accounting for 45.78% of the total SSRs. Adenine (A) monomeric repeats accounted for 54.04% (107/198) of the monomeric SSRs. In addition, 94 tandem repeats with ≥ 74% matches and lengths between 10 and 45 bp were identified in this genome (Table S3). There were 4,459 pairs of dispersed repeats with a length of ≥ 50 bp (Fig. 3B and Table S4), including 2,345 pairs of palindromic repeats and 2,114 pairs of forward repeats. The longest forward and backward repeats were 394,588 bp and 13,437 bp, respectively. However, no reverse repeat or complementary repeat was detected. In addition, the total lengths of the SSRs, tandem repeats, and dispersed repeats were 7,130 bp, 4,557 bp, and 1,941,444 bp accounting for 0.31%, 0.20%, and 84.78% of the mitogenome length, respectively.

Repetitive sequences that mediate genomic recombination may lead to multiple conformations in plant mitogenomes [40]. Specifically, there may be a secondary genomic structure mediated by repeat fragments R1 (394,588 bp, contig3 + contig9 + contig6), R2 (124,827 bp, contig5), and R3 (13,437 bp, contig9) (Table 2) in the *S. monacanthus* mitogenome. The recombination mediated by these three repeats was identified using a validated junction approach; the primer design and electrophoresis results are shown in Fig. S3, and detailed sequencing comparison results are shown in Fig. S4. In short, there are multiple potential recombination conformations in the *S. monacanthus* mitogenome.

**Table 2:** List of three repeated sequences mediating genomic recombination in the *S*. *monacanthus* mitogenome

Repeat Name	Repeat 1	Repeat 2	Repeat 3
Identities (%)	100	100	100
Length (bp)	394,588	124,827	13,437
Position-1	653,265-1,047,853	1,082,361-1,207,188	763,029–776,466
Position-2	1,895,431-2,290,019	1,578,808-1,703,635	1,847,779-1,861,216
E-value	0	0	0
Type	Forward	Forward	Palindromic

### RNA editing events in the PCGs

**Fig. 4:**
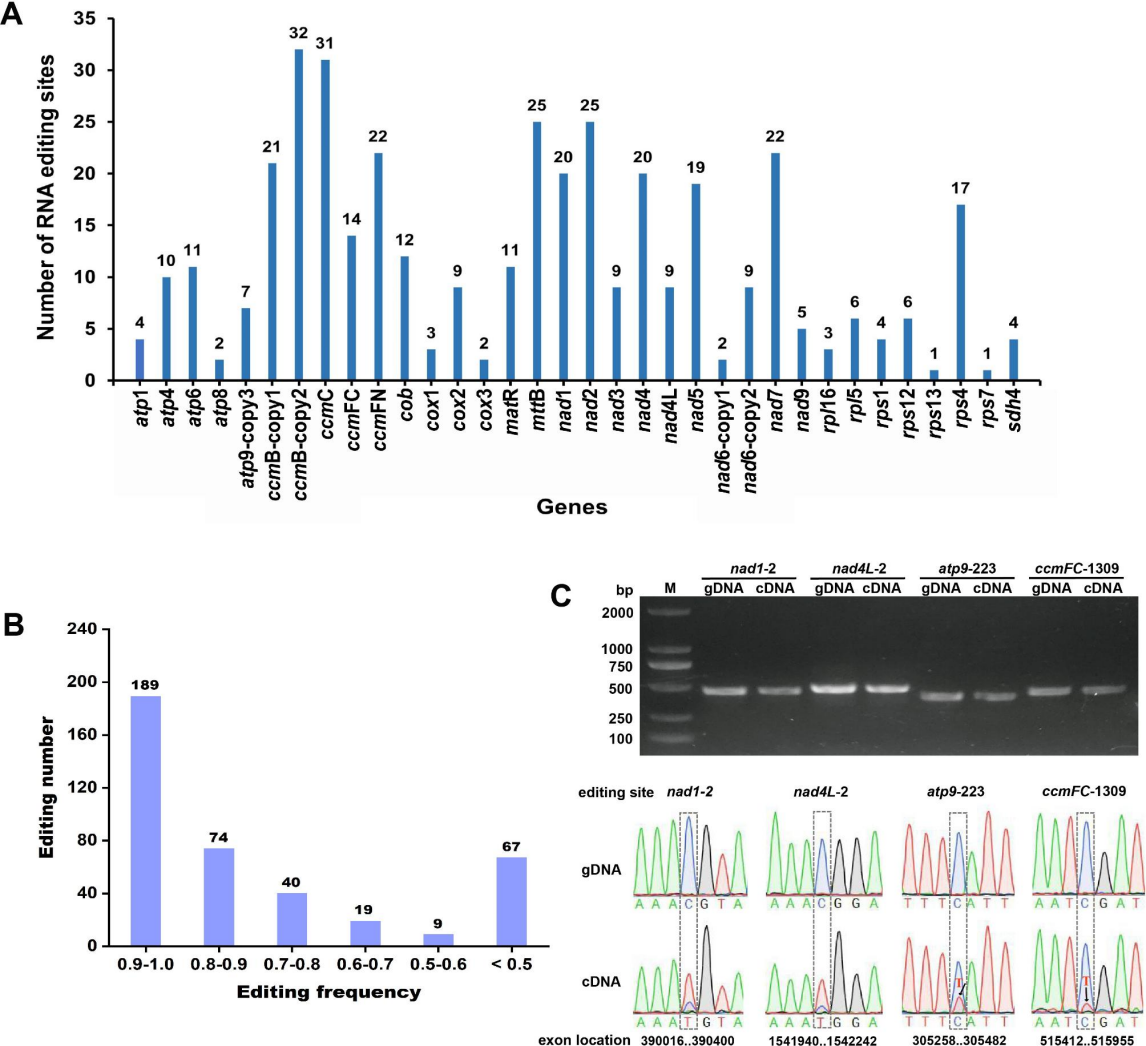
Editing number in PCGs (**A**), frequency distribution (**B**), and site-specific validation (**C**) of RNA-editing sites in *S. monacanthus*

There were a total of 398 RNA editing sites, and they were spread throughout the 32 PCGs of the *S. monacanthus* mitogenome. Each event was a C to U conversion (Fig. 4A and Table S5), and their editing frequencies were mostly above 0.80 (Fig. 4B). Among these, the highest number of RNA editing sites was in the *ccmB*-copy2 gene (No. 32), followed by that in the *ccmC* gene (No. 31). In addition, non-synonymous codon changes in the 356 RNA editing events mainly involved the following five amino acid changes: Ser to Leu (No. 87), Pro to Leu (No. 78), Ser to Phe (No. 44), Pro to Ser (No. 35), and Arg to Trp (No. 32).

In particular, we predicted that four editing sites were associated with the creation of start and stop codons in PCGs, that is, ACG (Thr) to AUG (Met) changes found in *nad1*-2 and *nad4L*-2, and CGA (Arg) to UGA (End) changes found in *atp9*-copy3-223 and *ccmFC*-1309. These four editing sites were validated by PCR products and Sanger sequencing comparison results (Fig. 4C and Supplementary File 1), where *atp9*-copy3-223 and *ccmFC*-1309 were edited with low frequency (the red line indicates base T in the transcription). However, its specific effects on the function and metabolism of mitochondria in plants are unknown.

### DNA transfer

**Fig. 5:**
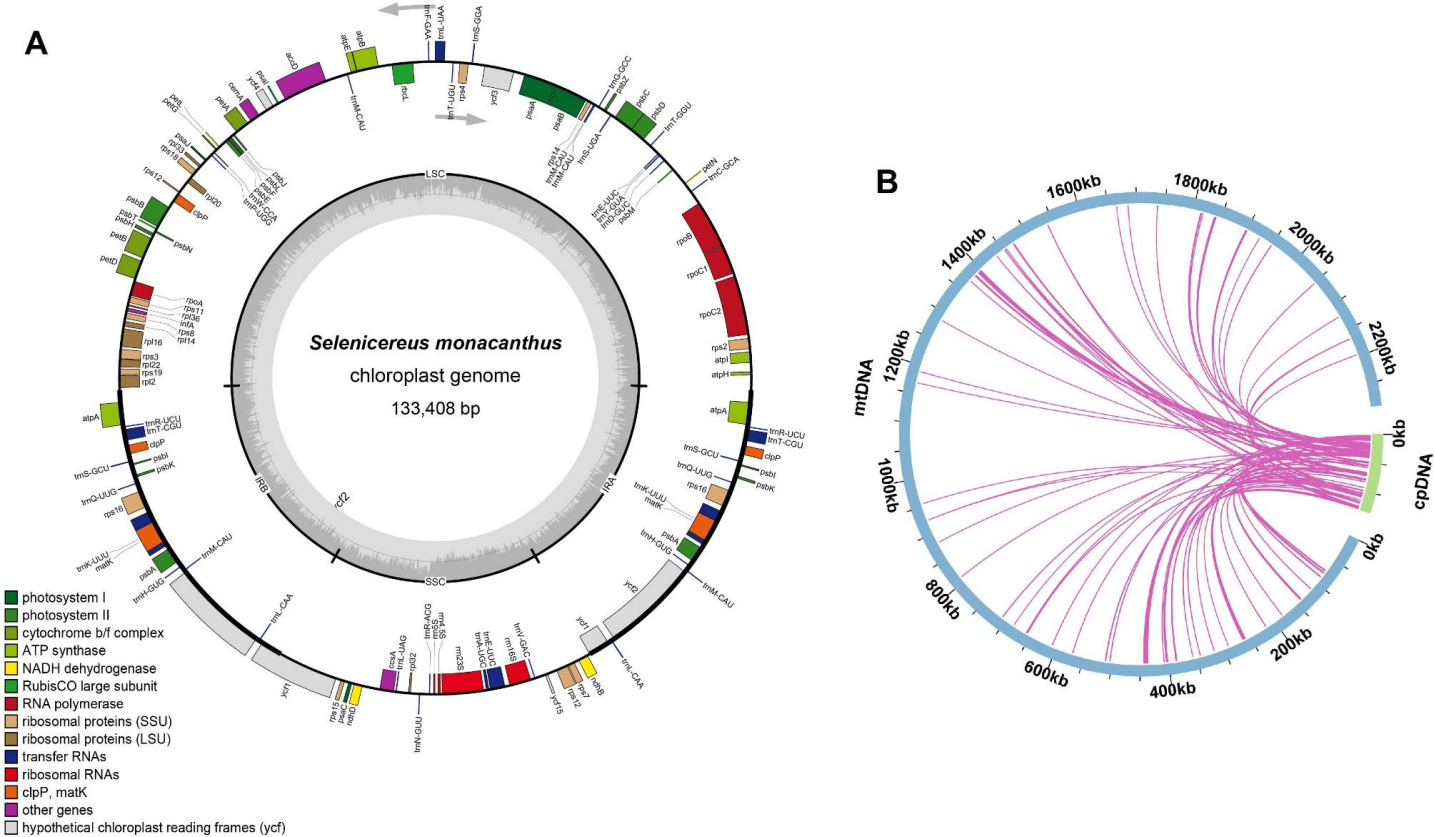
Chloroplast genome (**A**) and DNA transfer analysis (**B**) of *S. monacanthus*. The light blue and light green arcs represent the mitogenome and cp. genome, respectively. The purple lines between the arcs correspond to the homologous genomic fragments

Mitochondrial plastid DNAs (MTPTs) are plastid-derived DNA fragments found in the mitochondrial genome. In the present study, sequencing data were used to assemble the *S. monacanthus* chloroplast (cp.) genome, which was 133,408 bp in size (Fig. 5A). A total of 78 MTPTs were identified in the *S. monacanthus* cp. genome (Fig. 5B and Table S6), with a total length of 46,496 bp, accounting for 2.03% of the mitogenome length. There were 16 fragments with lengths greater than 1,000 bp, of which MTPT18 was the longest at 4,523 bp. Twenty-five complete genes were identified, including 14 PCGs (*atpA*, *atpB*, *atpE*, *psbA*, *psbD*, *psbE*, *psbF*, *psbJ*, *psbL*, *rpoC1*, *rps2*, *rps4*, *rps7*, and *ycf15*), and 11 tRNA genes (*trnD-GUC*, *trnF-GAA*, *trnH-GUG*, *trnN-GUU*, *trnM-CAU*, *trnR-ACG*, *trnR-UCU*, *trnS-GGA*, *trnT-CGU*, *trnV-GAC*, and *trnW-CCA*). In addition, 30 plastid gene fragments were identified among the homologous fragments. Detailed information on the DNA transfer fragments and gene annotations is presented in Table S6. However, these homologous genes are pseudogenized in mitochondria and do not exercise their normal functions [41], and their specific roles remain to be studied in depth in *S. monacanthus*.

### Genome evolution

**Fig. 6:**
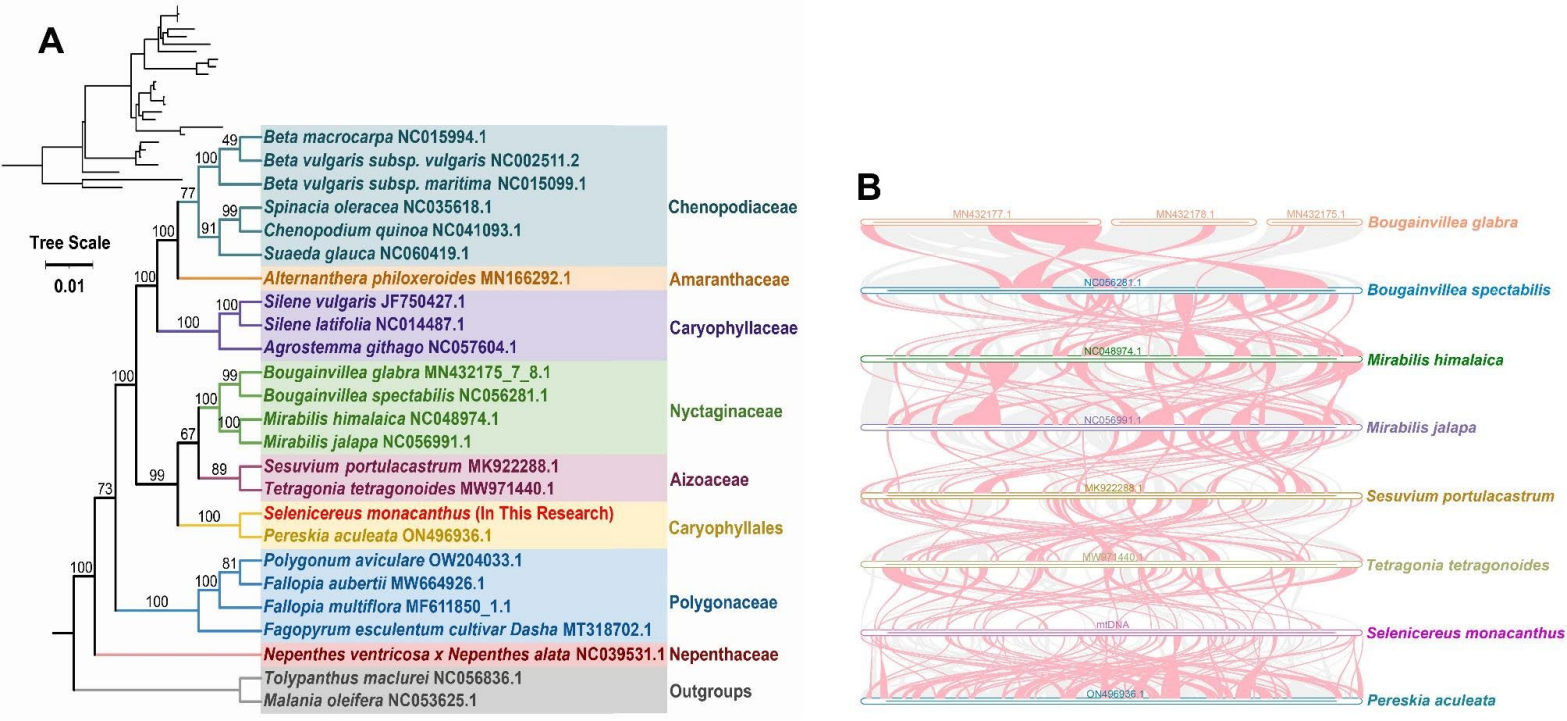
Phylogenetic analysis (**A**) and collinearity analysis (**B**) between *S. monacanthus* and related genera. (**A**) Bootstrap support values with different colors indicate different orders. (**B**) The pink, blue, dark green, dark purple, orange, gray, purple, and light blue lines represent *Bougainvillea glabra*, *Bougainvillea spectabilis*, *Mirabilis himalaica*, *Mirabilis jalapa*, *Sesuvium portulacastrum*, *Tetragonia tetragonoides*, *Selenicereus monacanthus*, and *Pereskia aculeata*, respectively. The red curved areas indicate regions where inversions occur and the gray areas indicate regions of good homology

Plant mitochondrial genomes commonly undergo a loss or gain of PCGs during evolution [13, 42]. Only 19 PCGs were common among *S. monacanthus* and 24 related genera used for phylogenetic analysis: *atp1*, *atp4*, *atp6*, *atp8*, *ccmB*, *ccmC*, *ccmFC*, *ccmFN*, *cox2*, *cox3*, *matR*, *nad1*, *nad2*, *nad3*, *nad5*, *nad6*, *nad7*, *nad9*, and *sdh4*. The phylogenetic tree showed that *S. monacanthus* was closely related to *P. aculeata* (Fig. 6A). Moreover, the topology based on the phylogeny of mitochondrial DNA coincided with the latest classification of the angiosperm phylogenetic group.

Collinear relationships between *S. monacanthus* and seven related species in Caryophyllales showed that 227 colinear blocks were detected between *S. monacanthus* and *P. aculeata* of the Cactaceae family; this accounted for 99.68% (513,522 bp) of the entire *P. aculeata* mitogenome but only 22.42% of the *S. monacanthus* genome (Fig. 6B and Table S7). Many homologous syntenic regions were detected between *S. monacanthus* and closely related species, however the length of these colinear blocks was short. Among the mitogenomes of these species, the inconsistent order of the colinear block arrangement implies that *S. monacanthus* may have undergone multiple genomic rearrangement events with closely related species.

## Discussion

### Size and genetic composition properties of the *S. monacanthus* mitogenome

This study combined short- and long-reads using a hybrid assembly strategy to assemble a high-quality *S. monacanthus* mitogenome, which is a full-length 2,290,019 bp ring-like molecule and one of the larger genomes, significantly larger than that of *P. aculeata* (515.2 kb) in the same family [38]. The diversity of GC content in plant mitogenomes reflects their adaptive consequences [11, 43]. The GC content in the mitogenome of *S. monacanthus* was 43.37%. This was relatively less different from that of other terrestrial plants (23.9–50.5%). Gene transfer is the main pathway for the loss of mitogenome PCGs in plants, most of the transferred genes are ribosomal protein-encoding genes, with only some respiratory chain-related genes transferred to the nucleus during evolution (*rps2*, *rps11*, and *rps19*) [42]. *L. tulipifera* is a “fossilized” plant whose mitogenome evolved very slowly, retaining 41 PCGs from the ancestral angiosperms [39]. It was hypothesized that *S. monacanthus* lost at least nine PCGs through gene loss or transfer events during evolution (*rpl2*, *rpl10*, *rps2*, *rps3*, *rps10*, *rps11*, *rps14*, *rps19*, and *sdh3*). In addition, gene distribution density of the *S. monacanthus* mitogenome was very low. The coding sequence was highly conserved and its coding region accounted for only 2.17% of the full genome length; this was significantly lower than that of *L. tulipifera* (excluding *cis*-spliced introns, 7.9%) [39] and *Populus simonii* (8.25%) [44].

### Repeated sequences are exceptionally abundant in the *S. monacanthus* mitogenome

Repetitive sequences are abundant in the mitogenome and play important roles in the evolution of plant adaptation, regulation of gene expression, and variation in epistatic traits [45]. This study obtained 616 SSRs from the *S. monacanthus* mitogenome. This provides a large number of reference loci for further species identification and genetic evolution. Moreover, mitogenome-coding sequences have a slower evolutionary rate than chloroplast and nuclear genes [11]. Therefore, the development of mitogenome molecular markers is more accurate for species identification. Numerous studies show that dispersed repeats can affect plant phenotypic traits through the expression of regulatory genes [26–28, 46]. A total of 4,459 pairs of dispersed repeats ≥ 50 bp in length were detected in the *S. monacanthus* mitogenome; they mainly existed in the intergenic region and accounted for 84.78% of the mitogenome. This is one of the largest numbers of repeats identified in all other published mitogenic sequences. This implies that dispersed repeats may play an important role in genome expansion and gene regulation, and also provides scientific reference information for further study of their effects on agronomic traits in *S. monacanthus*.

### *S. monacanthus *has multiple conformations

Plant mitogenomes are commonly characterized by rearrangements that are important in promoting genome evolution and enriching genetic diversity [8, 40]. Moreover, the frequency of repeated recombination is related to the length of the repeat sequence and the characteristics of the species. In general, long repetitive sequences (> 1000 bp) with high similarity are more likely to recombine [14]. We predicted and confirmed the existence of genomic recombination mediated by three repeats in the *S. monacanthus* mitogenome based on long-read mapping results: R1 (394,588 bp), R2 (124,827 bp), and R3 (13,437 bp). However, the presence of short-repeat sequence-mediated recombination could not be determined, and this needs to be supported by high-sequencing depth data. This implies possible differentiation within the mitogenome of the genus *Selenicereus* and provides useful information to gain insight into the evolution of genomes in *S. monacanthus* and closely related genera.

### RNA editing events are prevalent in the PCGs of the *S. monacanthus* mitogenome

RNA editing is an important post-transcriptional regulatory mechanism and a biological process prevalent in higher plant mitochondria where single-base conversion is the most prevalent RNA editing event [47, 48]. Our study showed that all 32 PCGs of the *S. monacanthus* mitogenome underwent RNA editing events. Each event was a single-base edit (C to U) that mostly resulted in amino acid conversion. This may allow the genes to acquire new structures and functions. RNA editing is closely related to cytoplasmic male sterility. Stop codon editing shortens the *orf77* chimeric open reading frame associated with male sterility in maize and eventually leads to pollen abortion [49]. Meanwhile, the change from CGA (Arg) to UGA (End) at position 223 of *atp9* ensured normal synthesis of this polypeptide in the Yunnan purple rice maintenance line (YingxiangB) [50], whereas no RNA editing occurred at this site in the sterile line (YingxiangA). Plant mitochondrial RNA editing can introduce new start codons. For example, the conversion of ACG (Thr) to AUG (Met) is the starting point for the transcription of the *nad1* gene in wheat and the *cox1* gene in tomato and potato [51, 52]. Usually after generating new start and stop codons, it encodes proteins that are more conserved, and higher homology with corresponding proteins from other species allows for better expression of genes in mitochondria [47]. This study further revealed that the start or stop codons of four genes were generated by RNA editing events in the *S. monacanthus* mitogenome, i.e., new start codons by loci *nad1*-2 and *nad4L*-2, while new stop codons by *atp9-*copy3-223 and *ccmFC*-1309. However, their effects on mitochondria and plants require further investigation.

### Gene transfer and gene loss are common during *S. monacanthus* evolution

Plant mitogenomes can integrate exogenous or migratory DNA sequences by intracellular or horizontal transfer [13, 20]. This leads to the accumulation of large amounts of repetitive sequences and the gain/loss of large DNA fragments in the genome [8, 53]. The introduced genes usually degenerate into pseudogenes [54]. This study found 78 homologous fragments of the chloroplast genome with a total length of 46,496 bp in the *S. monacanthus* mitogenome. The same phenomenon was observed in *Mangifera indica* [55], *Taraxacum mongolicum* [56], and *P. aculeata* [38]. However, no opposite sequence migration was observed in *S. monacanthus*. Fourteen PCGs and eleven tRNAs were identified among the homologous sequences of *S. monacanthus*, and most of the remaining gene sequences lost their integrity. Evolutionary analysis and comparison revealed that only 19 PCGs were identical among 25 closely related species, and the genome sequences of *S. monacanthus* and seven closely related species of the same Order were highly inconsistent in terms of genome sequence, even for the more closely related *P. aculeata* of the same family. This suggested that the species may have undergone frequent genome recombination events during evolution. This study further confirms the idea of mitogenomic gene transfer or loss and provides an effective way to deeply explore the evolutionary history of *S. monacanthus* and closely related species.

## Conclusions

This is the first published assembly of the *S. monacanthus* mitogenome, which is 2,290,019 bp in length. It encoded 59 unique genes that accounted for only 2.17% of the total length. Several dispersed repeats, plastid DNA fragments, and RNA editing events were identified in this genome, and multiple potential conformations may exist since the three repeats mediate recombination. Evolutionary analysis suggested that multiple genomic recombination and gene loss events may have occurred in *S. monacanthus* during its evolution. This study provided important information for an in-depth study of the evolutionary history and molecular breeding of *S. monacanthus*. Further, the genome of *S. monacanthus* can also be used as a reference genome for other *Selenicereus* species.

## Materials and methods

### Plant material and sequencing

The pitaya plant (Hong long 1) was cultivated at the National Agricultural Science and Technology Park in Lhasa, Tibet Autonomous Region, China (location: 91°2’8’’E, 29°38’15’’N; altitude: 3650 m). The young shoots were harvested, immediately frozen in liquid nitrogen, and stored at -80 °C in an ultra-low temperature refrigerator (Qingdao Aucma Co., Ltd, Qingdao, China). DNA and RNA were extracted from the epidermal tissue of pitaya shoots using the TianGen Super Plant Genomic DNA Kit and the RNAprep Pure Plant Kit (Polysaccharides & Polyphenolics-rich) (Beijing, China), respectively. The quality of the DNA and RNA was checked using a NanoDrop One Microvolume UV-Vis Spectrophotometer (Thermo Fisher Scientific, Massachusetts, USA) and sent to Wuhan Benagen Tech Solutions Co., Ltd. (Wuhan, China) for sequencing. Short-reads, long-reads, and long non-coding RNA (lncRNAs) were sequenced using a DNBSEQ-T7 Genetic Sequencer (Shenzhen Huada Intelligent Technology Co., Ltd., Shenzhen, China), Nanopore PromethION sequencer (Oxford, UK), and MGISEQ-2000 sequencing platform (Shenzhen, China), respectively. Fastp v0.21.0 [57], NanoFilt v2.8.0 [58], and SOAPnuke v2.0 [59] were used to filter short-, long-, and lncRNA raw reads, respectively.

### Mitogenome assembly

The assembly of long reads from the sequencing data was performed using Flye software [60] to obtain graphical results in GFA format [61]. Subsequently, the BLASTN program was used to identify contig fragments containing the mitogenome with the parameter “-evalue 1e-5 -outfmt 6 -max_hsps 10 -word_size 7 -task blastn-short,” using the *Arabidopsis thaliana* genome as a query sequence. The short- and long-read data were then compared to the mitogenome contigs using BWA v0.7.17 [62], and the well-matched reads were filtered and exported for subsequent assembly. Finally, the hybrid assembly was implemented to obtain the complete mitogenome of *S. monacanthus* using Unicycler v0.4.7 (The University of Melbourne, Victoria, Australia) with the parameter “--kmers 57,67” [63].

### Gene annotation and codon preference analysis

The protein-coding genes (PCGs) of the *S. monacanthus* mitogenome were annotated using Geseq v2.03 (https://chlorobox.mpimp-golm.mpg.de/ geseq.html) [64] with the mitogenomes of *A. thaliana* (NC_037304) and *L. tulipifera* (NC_021152.1) used as references. tRNAscan-SE v2.0.11 was used to annotate tRNA genes [65] and BLASTN v2.13.0 was used for rRNA gene annotation [66]. The errors were manually corrected using Apollo v1.11.8 [67]. The PCGs were extracted using PhyloSuite v1.2.2 [68] and used for codon preference analysis using Mega v7.0.26, with relative synonymous codon usage (RSCU) values calculated [69]. An RSCU value > 1 indicates that the codon is preferentially used by amino acids, whereas an RSCU value < 1 indicates the opposite trend.

### Repeat element identification

The SSRs in the *S. monacanthus* mitogenome were identified using MISA v2.1 (https://webblast.ipk-gatersleben.de/misa/) [70] with the parameter “1–10 2–5 3–4 4 − 3 5 − 3 6 − 3”. Tandem repeats were recognized using TRF v4.09 (https://tandem.bu.edu/trf/trf.unix.help.html) with the parameter “2 7 7 80 10 50 500 -f -d -m” [71]. Dispersed repeats were detected using REPuter (https://bibiserv.cebitec.uni-bielefeld.de/reputer/) [72] with the repeat size ≥ 50 bp. The results were visualized using Excel 2021 and Circos 0.69-9 [73].

### Repeat-mediated recombination validation

Unicycler was used to derive the sequences at the branching nodes and map them to long reads; those supported by longer reads were prioritized. The correctness of the assembly was verified by extracting each pair of repetitive sequences and using the 500 bps upstream and downstream of the sequence as a reference. We then designed primers for the four paths of the double bifurcating structure using Primer-BLAST (https://www.ncbi.nlm.nih.gov/tools/primer-blast) (Table S8), and the authenticity of the interface sequences was verified by PCR amplification and Sanger sequencing [12, 56]. The amplification was performed using an Applied Biosystems real-time PCR instrument (Thermo Fisher Scientific, Massachusetts, USA) in a total volume of 50 µL, including 2 µL of DNA template, 2 µL each of upstream and downstream primer (10 µmol/L), 25 µL of 2× Rapid Taq Master Mix (Vazyme Biotech Co., Ltd., Nanjing, China), and 19 µL of ddH_2_O. The cycling procedure included pre-denaturation at 95 °C for 3 min, followed by 35 cycles of 95 °C for 15 s (denaturation), 55 °C for 15 s (annealing), and 72 °C for 30 s (extension), with a final extension at 72 °C for 15 min.

### RNA editing site prediction and validation

The transcripts from the *S. monacanthus* mitogenome were obtained from transcriptomic data by filtering, mapping to mitochondrial DNA sequences using TopHat2 with mismatches of 7 [74], and further comparison of DNA and RNA sequences using REDItools v2.0 [75] to identify the potential RNA editing events in mitogenome PCGs, with a coverage depth ≥ 100× and editing frequency ≥ 0.10. Primers for specific editing sites were designed using Primer-BLAST software (Table S9). RNA was reverse transcribed into cDNA using a HiScript III 1st Strand cDNA Synthesis Kit (Vazyme, Nanjing, China). PCR amplification was performed using gDNA and cDNA as templates, and the validation method is the same as in the above section. The amplified products were compared by Sanger sequencing.

### Homologous DNA analysis

The GetOrganelle v1.7.7.0 software [76] was used to extend the short reads of *S. monacanthus* chloroplast genome, the SPAdes software in Unicycler was used to assemble the extended reads to form a unitig map with the parameters “-R 15 -k 21,45,65,85,105 -F embplant_pt”, and the long reads were utilized to solve the bifurcation structure in the unitig graph using Unicycler. Annotation was performed using CPGAVAS2 (http://47.96.249.172:16019/analyzer/annotate) [77] and the results were corrected using CPGView [78]. Homologous fragments of the chloroplast and mitochondrial genomes of *S. monacanthus* were analyzed using BLASTN [66] with an e-value of 1*e*-6 and a word size of 7.

### Evolution analysis

The mitogenomes of twenty-four species closely related to *S. monacanthus* were downloaded from the NCBI (Table S10), with *Malania oleifera* (NC_053625.1) and *Tolypanthus maclurei* (NC_056836.1) (MK431827.1) set as outgroups. PhyloSuite software was used to extract the common genes [68], with MAFFT v7.505 used for multiple sequence alignment [79]. Phylogenetic analysis was performed using IQ-TREE v1.6.12 with the “GTR + F + I + I + R2” model [80], and the maximum likelihood tree was visualized using iTOL v6 (https://itol.embl.de/). The mitogenomes of *S. monacanthus* and seven closely related species in the same Order (Caryophyllales) were compared and analyzed using the BLAST program. Homologous sequences ≥ 500 bp in length were retained as conserved co-linear blocks, and the Multiple Synteny Plot was plotted using the source program of MCscanX [81].

### Electronic supplementary material

Below is the link to the electronic supplementary material.

Supplementary Material 1

Supplementary Material 2

Supplementary Material 3

Supplementary Material 4

Supplementary Material 5

Supplementary Material 6

Supplementary Material 7

Supplementary Material 8

Supplementary Material 9

Supplementary Material 10

Supplementary Material 11

## Data Availability

The mitogenome sequence data of *S. monacanthus* are available in NCBI Nucleotide Database under the GenBank accessions: OQ835513. The BGI and Nanopore sequencing data of *S. monacanthus* have been deposited in the Figshare platform: doi:10.6084/m9.figshare.22350940 and doi:10.6084/m9.figshare.22350505. The raw transcriptome sequencing data of *S. monacanthus* have been submitted to the Sequence Read Archive (SRA) repository under SRR24044980. The mapping results (BAM files) were uploaded to the figshare platform: doi:10.6084/m9.figshare.22650259.

## Abbreviations

PCG: protein coding gene
RSCU: relative synonymous codon usage values
SSR: simple sequence repeat
MTPT: mitochondrial plastid sequence
lncRNA: long non-coding RNA
