# Supplementary figures and images for: Complete mitogenome assembly of Selenicereus monacanthus revealed its molecular features, genome evolution, and phylogenetic implications
Source: BMC Plant Biol. 2023 Nov 4;23:541. doi: 10.1186/s12870-023-04529-9 (PMC10625231; doi:10.1186/s12870-023-04529-9)

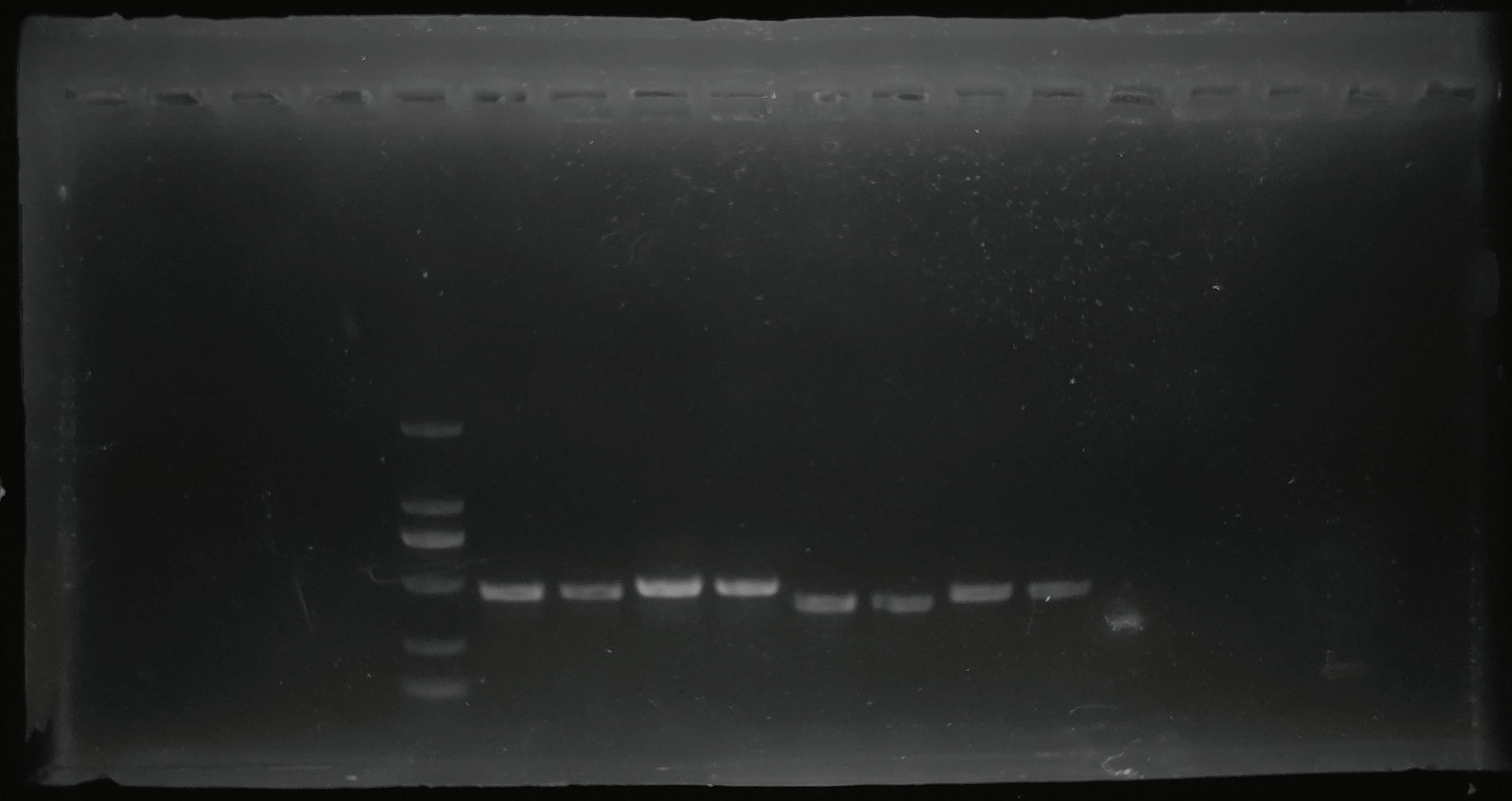

Supplement: Supplementary file 1 — Supplementary Material 1 [file 12870_2023_4529_MOESM1_ESM.jpg]

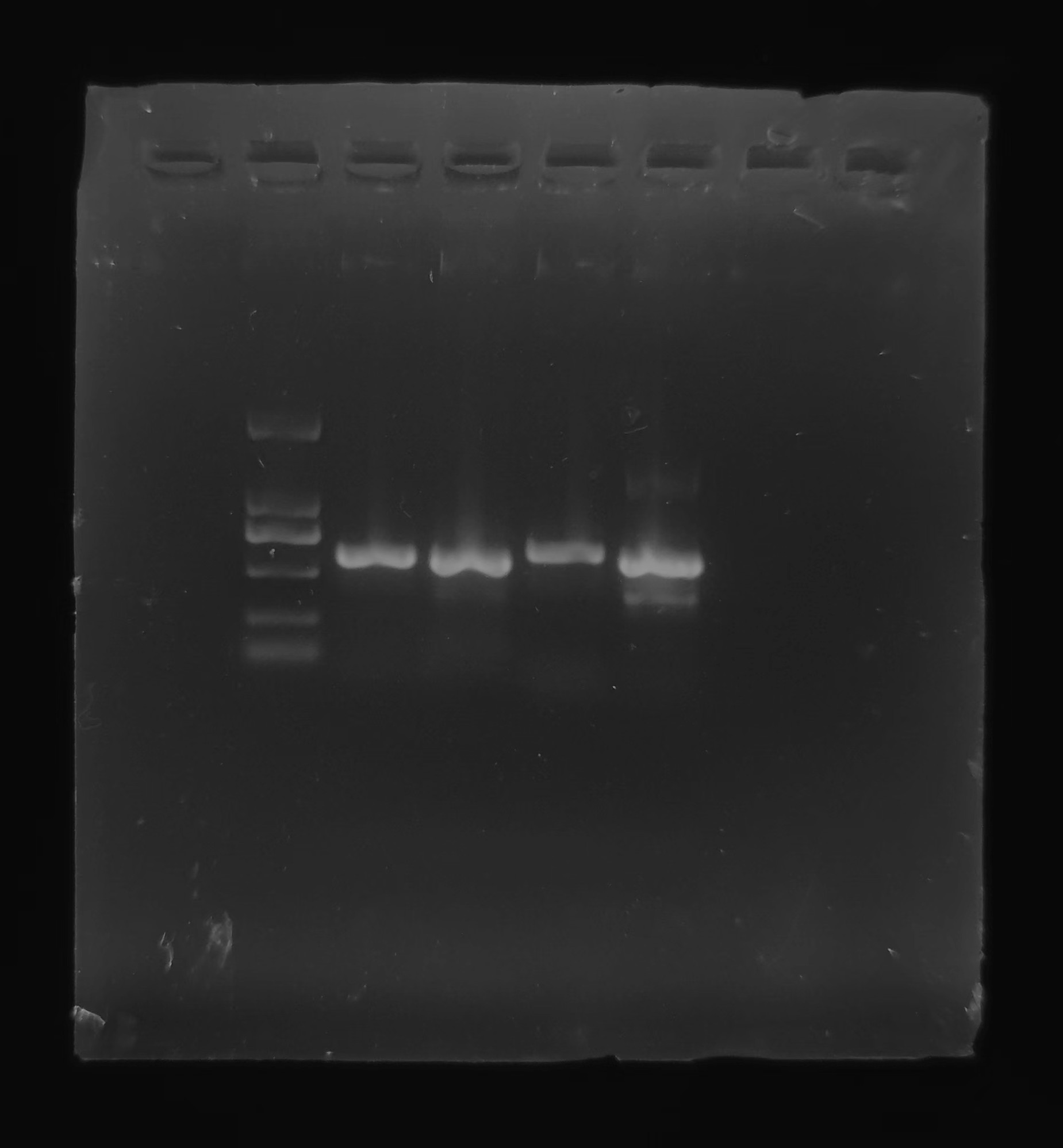

Supplement: Supplementary file 2 — Supplementary Material 2 [file 12870_2023_4529_MOESM2_ESM.jpg]

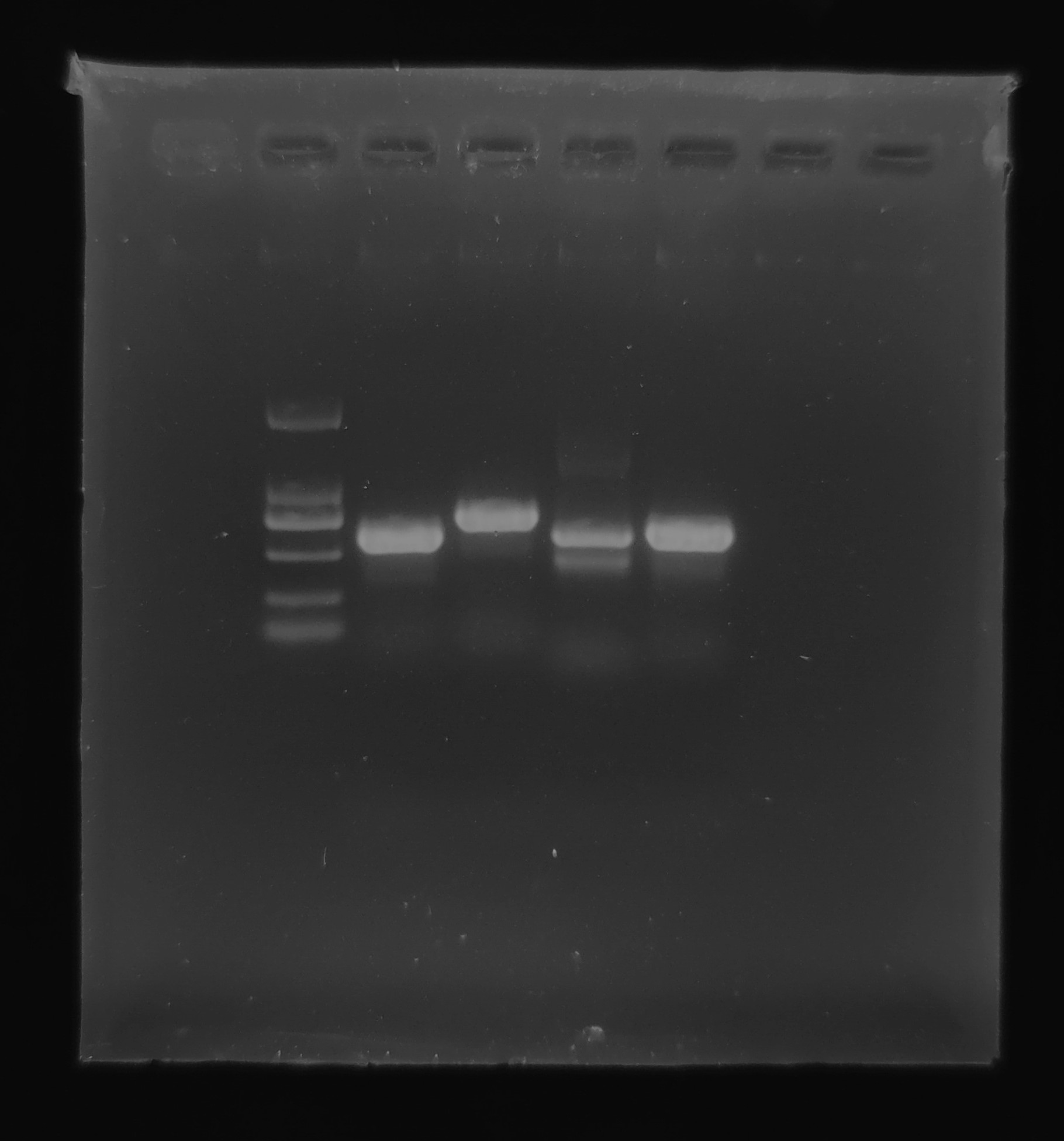

Supplement: Supplementary file 3 — Supplementary Material 3 [file 12870_2023_4529_MOESM3_ESM.jpg]

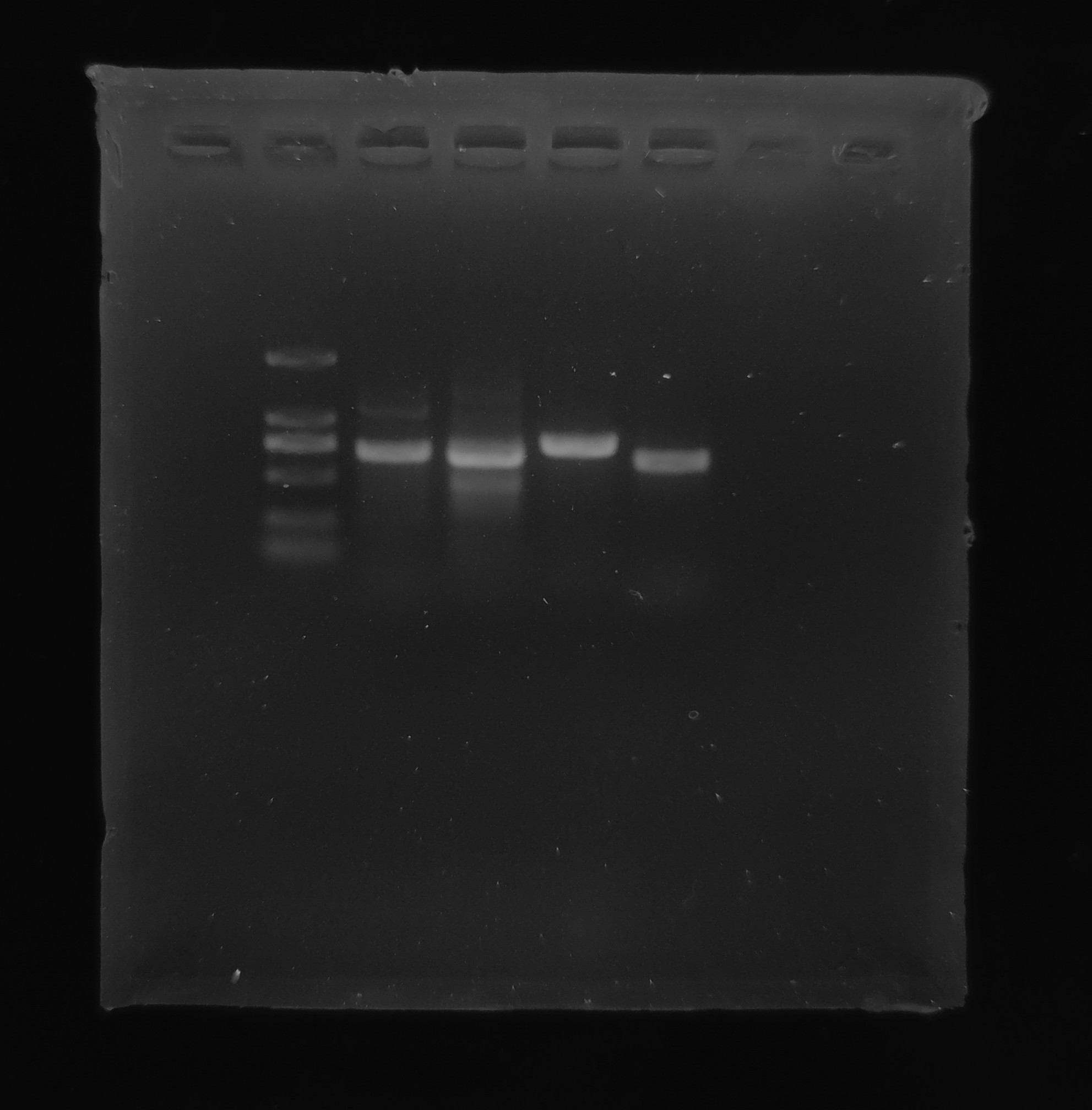

Supplement: Supplementary file 4 — Supplementary Material 4 [file 12870_2023_4529_MOESM4_ESM.jpg]

Supplementary 2. gDNA and cDNA sequence comparison of editing site nad4L-2

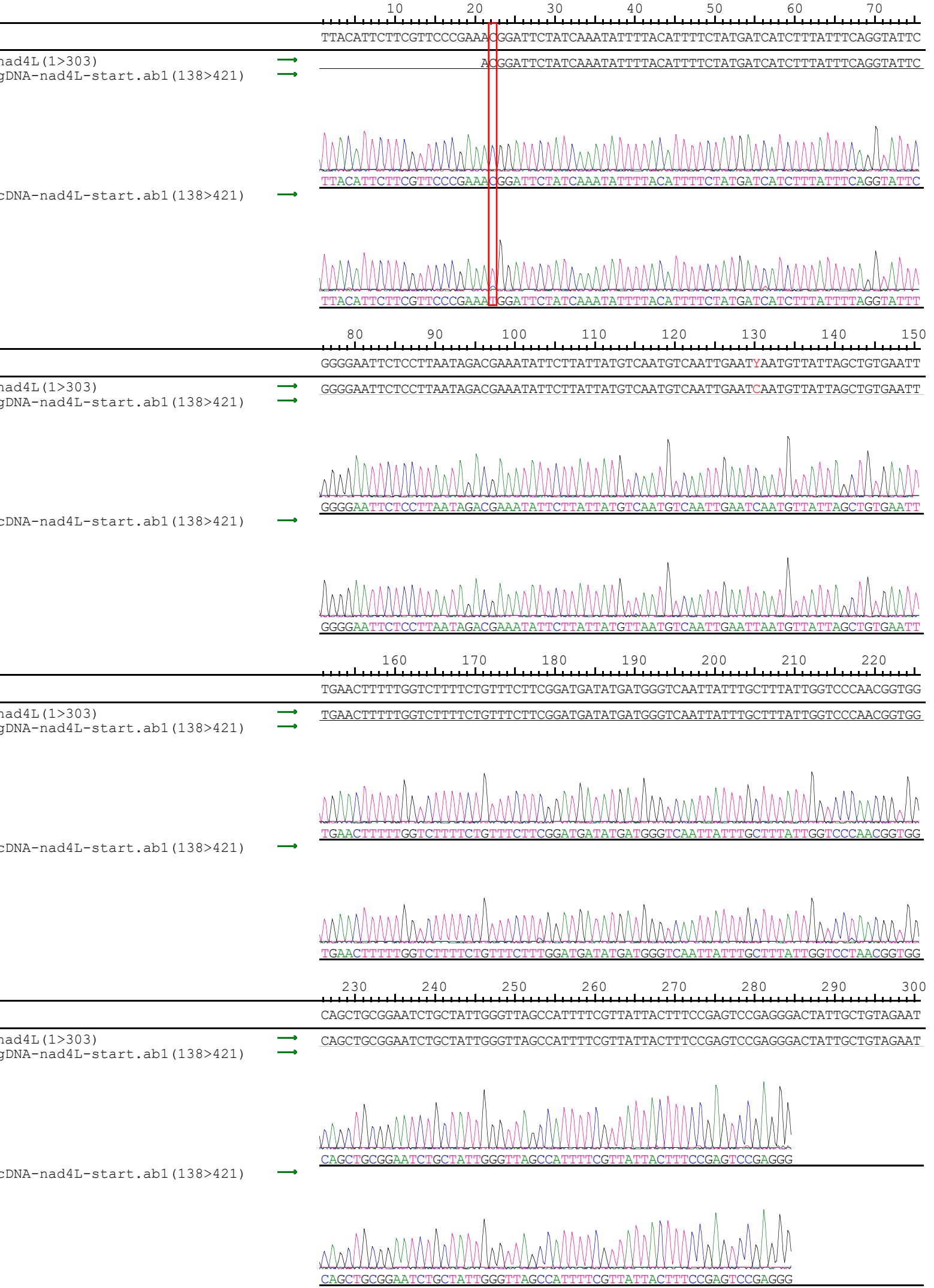

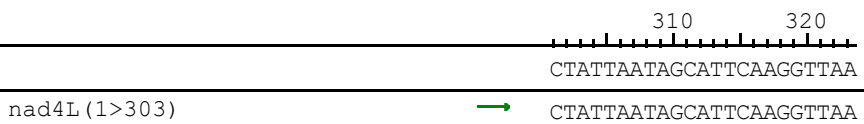

Supplement: Supplementary file 8 — Supplementary Material 8 [file 12870_2023_4529_MOESM8_ESM.pdf]

# Supplementary 3. gDNA and cDNA sequence comparison of editing site atp9-223

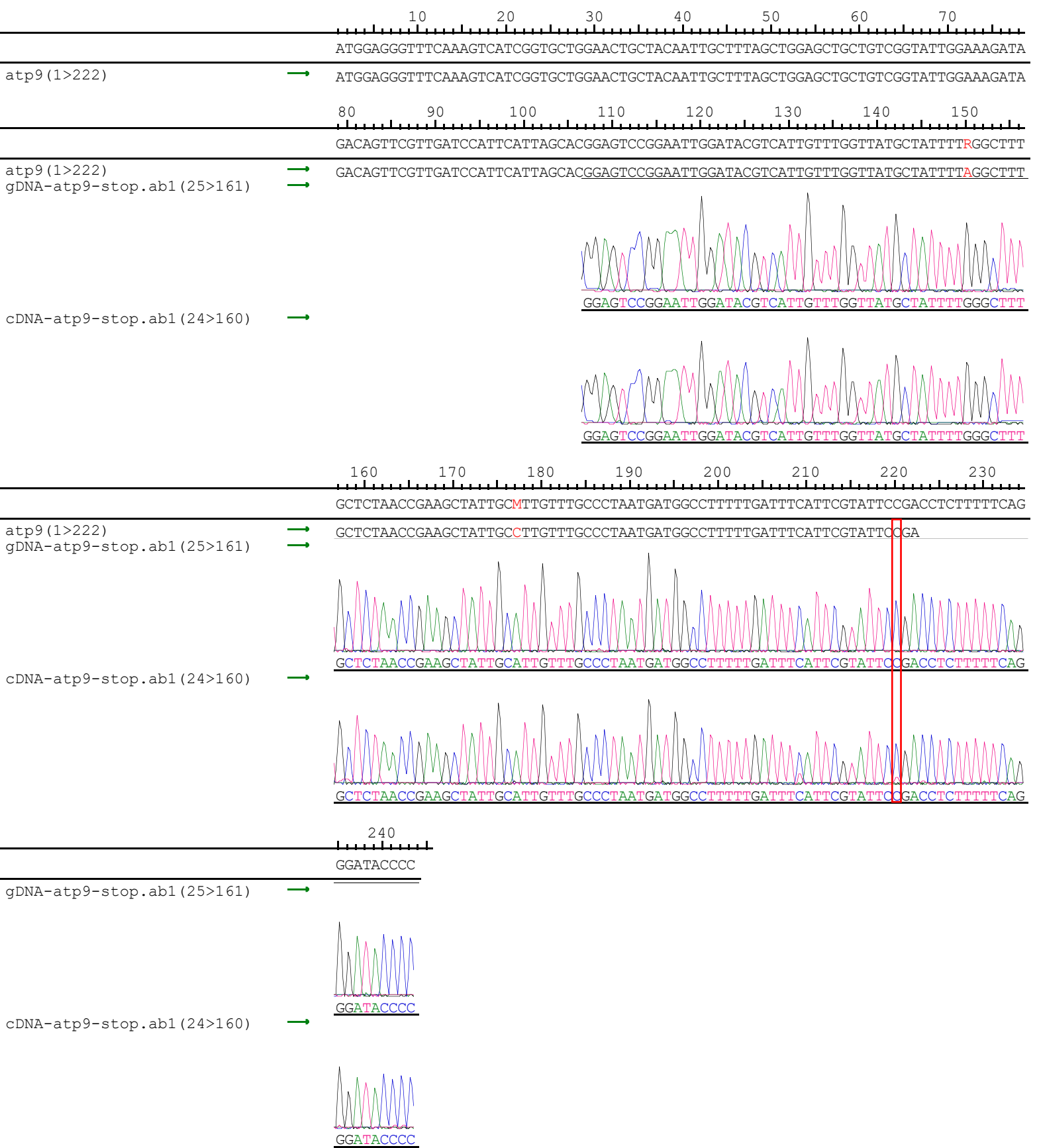

Supplement: Supplementary file 9 — Supplementary Material 9 [file 12870_2023_4529_MOESM9_ESM.pdf]
