# Supplementary material for: Complete mitogenome assembly of Selenicereus monacanthus revealed its molecular features, genome evolution, and phylogenetic implications
Source: BMC Plant Biol. 2023 Nov 4;23:541. doi: 10.1186/s12870-023-04529-9 (PMC10625231; doi:10.1186/s12870-023-04529-9)
Supplement: Supplementary file 5 — Supplementary Material 5 [file 12870_2023_4529_MOESM5_ESM.docx]

# Supplementary Figures


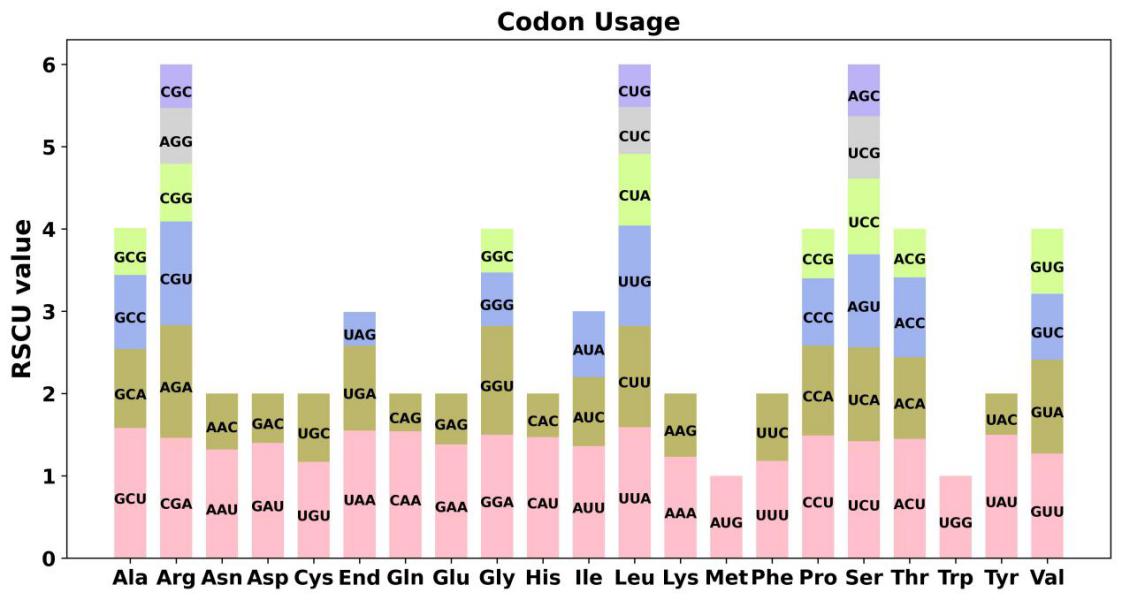


**Figure S1**. Codon usage preference of protein-coding genes in the *Selenicereus monacanthus.*


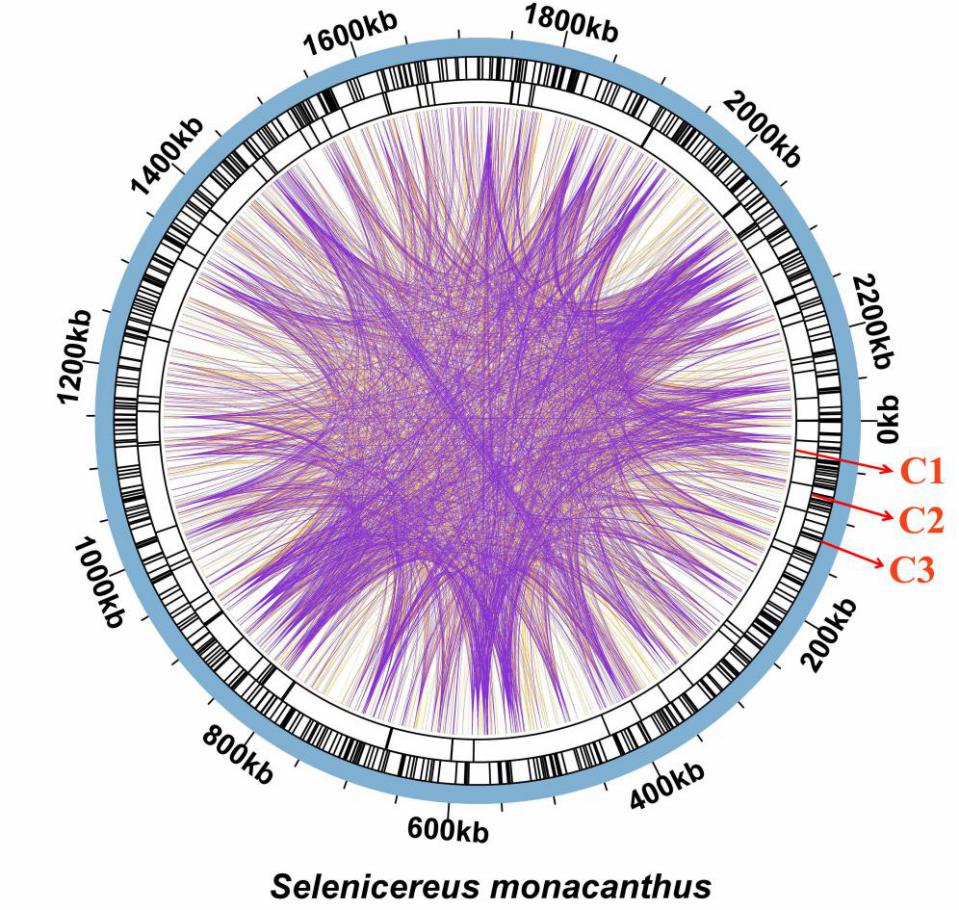


**Figure S2**. Repeats analysis of the mitochondrial genome in the *Selenicereus monacanthus.*

The color line on the C1 circle connects two repeated dispersed repeat. The purple and yellow lines represent palindromic repeat and forward repeats, respectively. The black line on the C2 circle represent tandem repeats. The black line on the C3 circle simple sequence repeats.


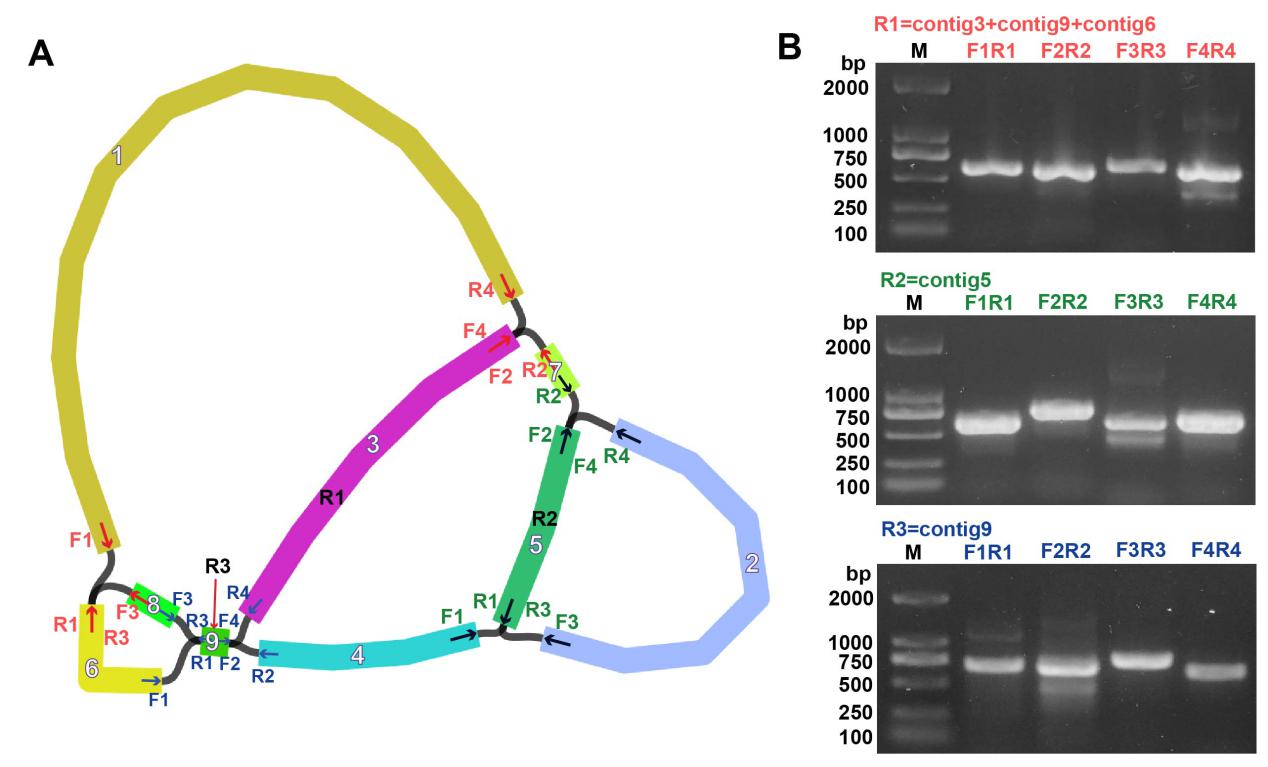


**Figure S3**. The recombination structure prediction (**A**) and verification (**B**) in *Selenicereus monacanthus*.


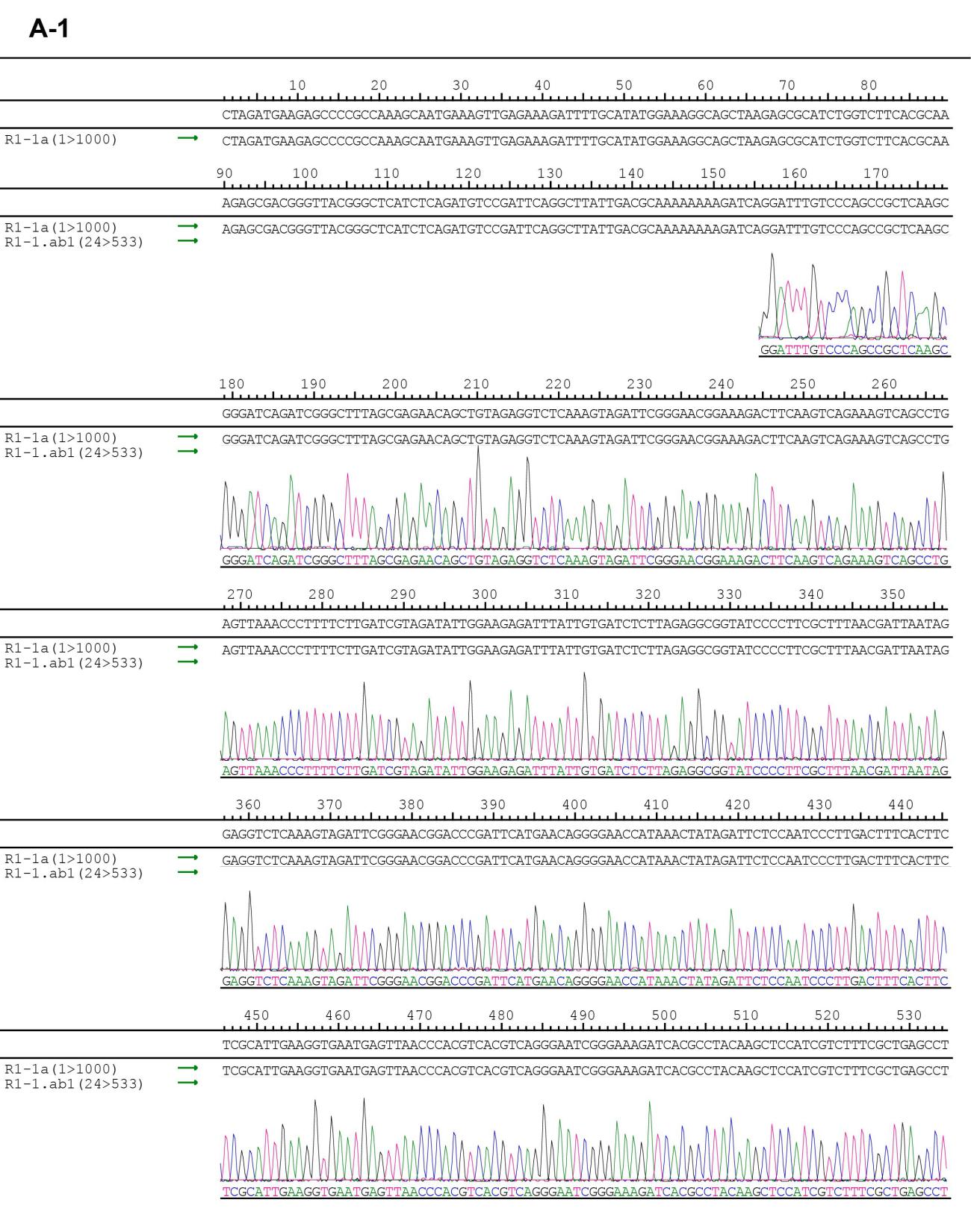


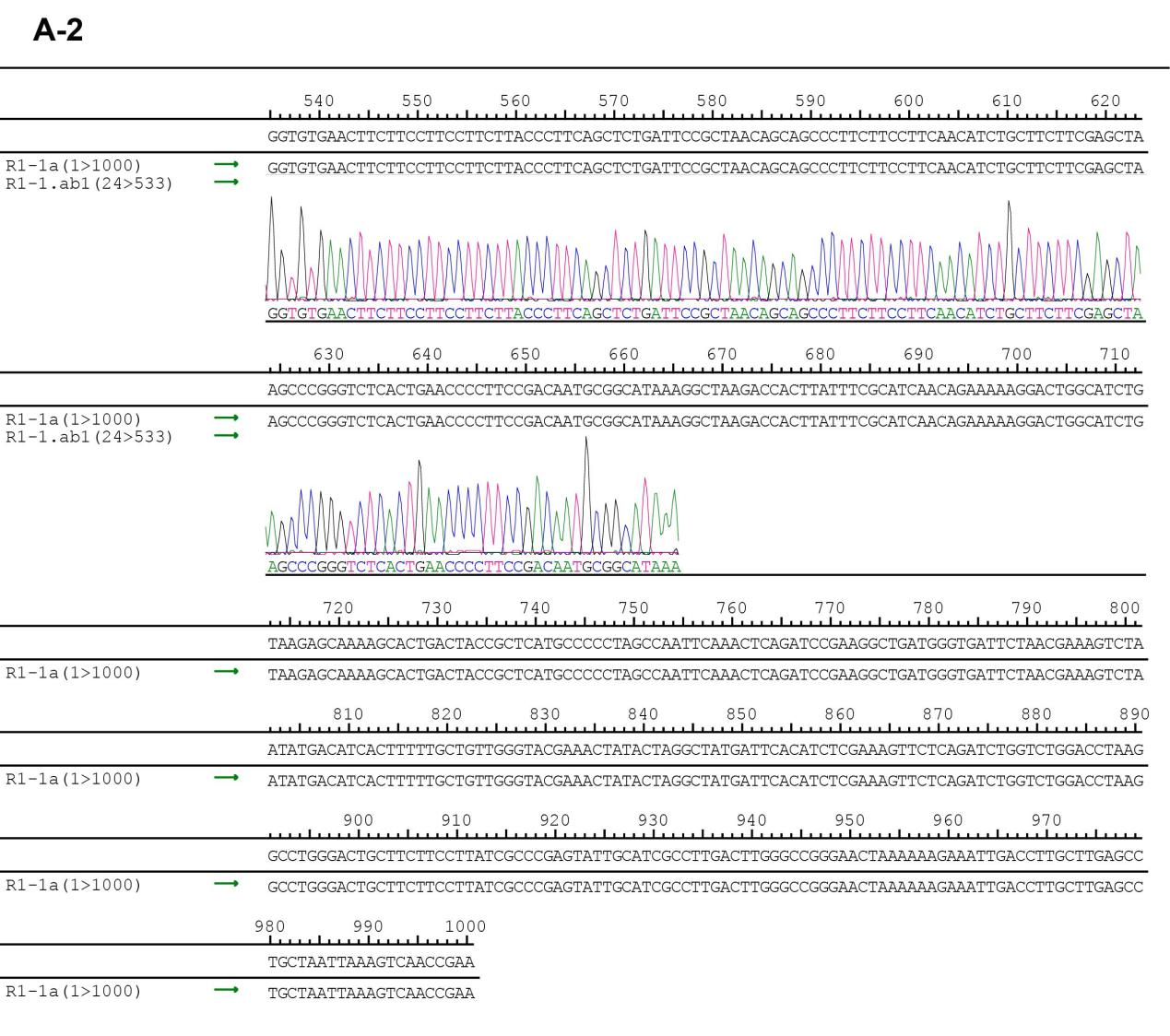


**Figure S4**. Boundary verification of repeated fragment (A: R1-1).


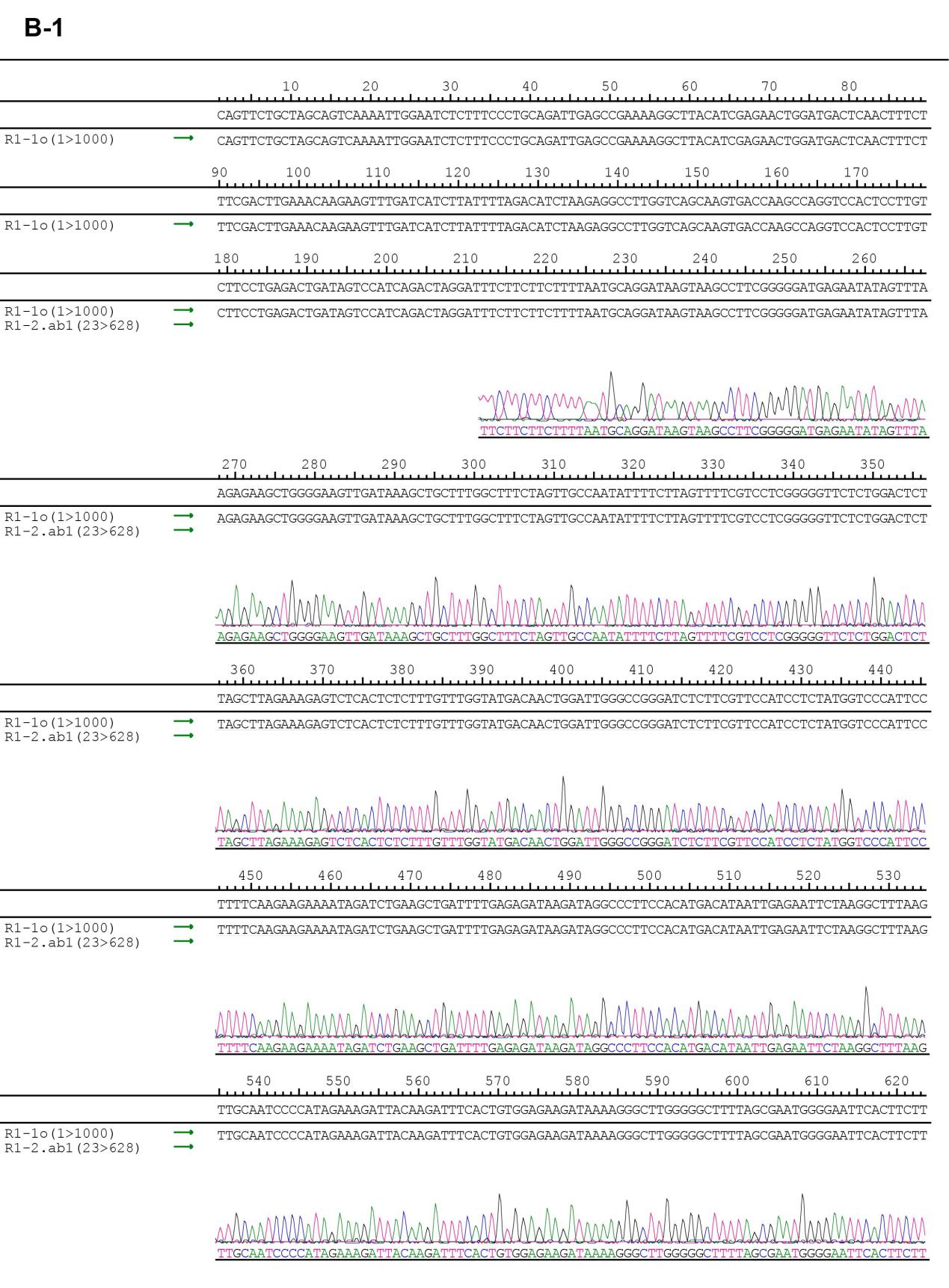


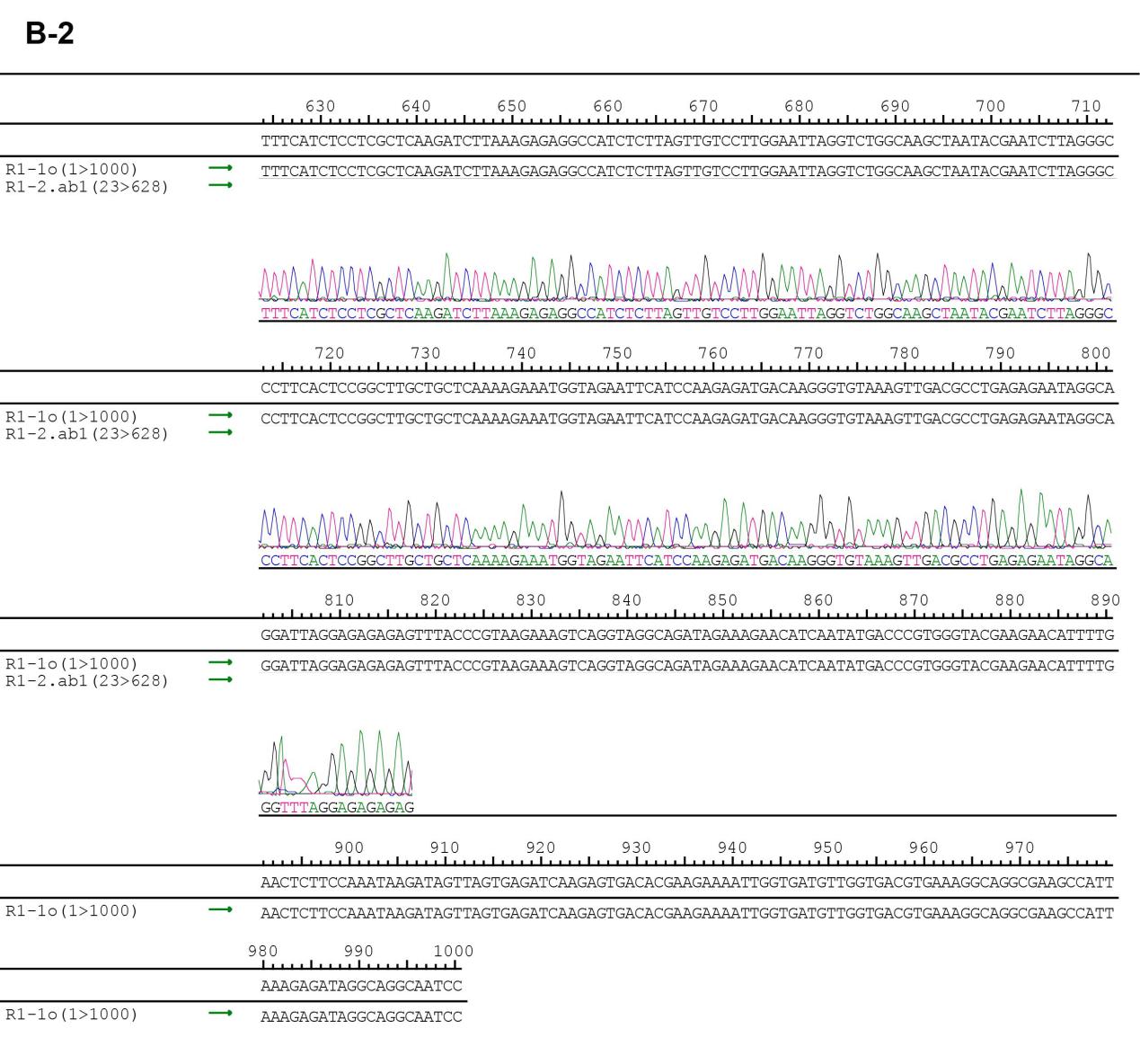


**Figure S4**. Boundary verification of repeated fragment (B: R1-2).


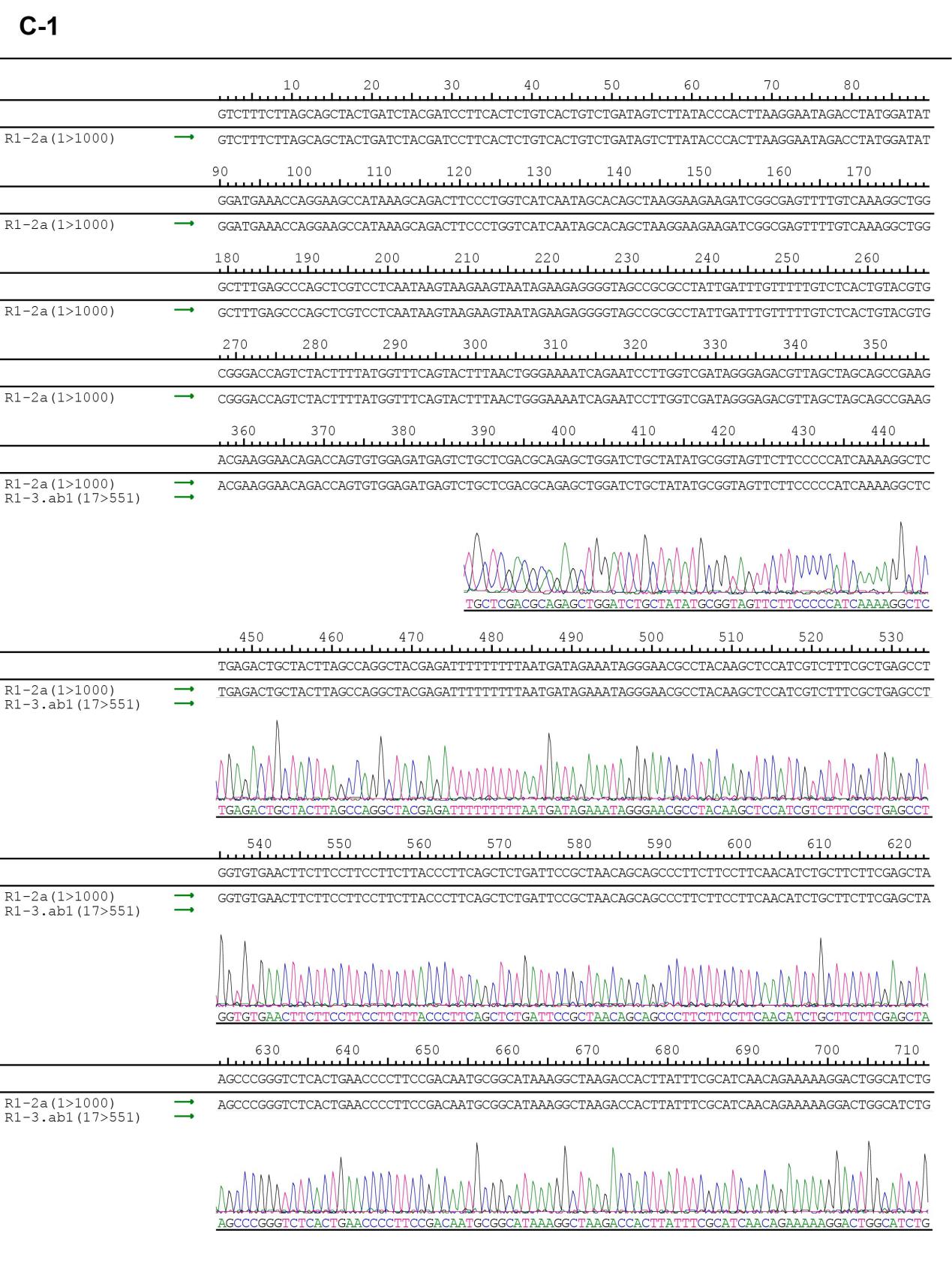

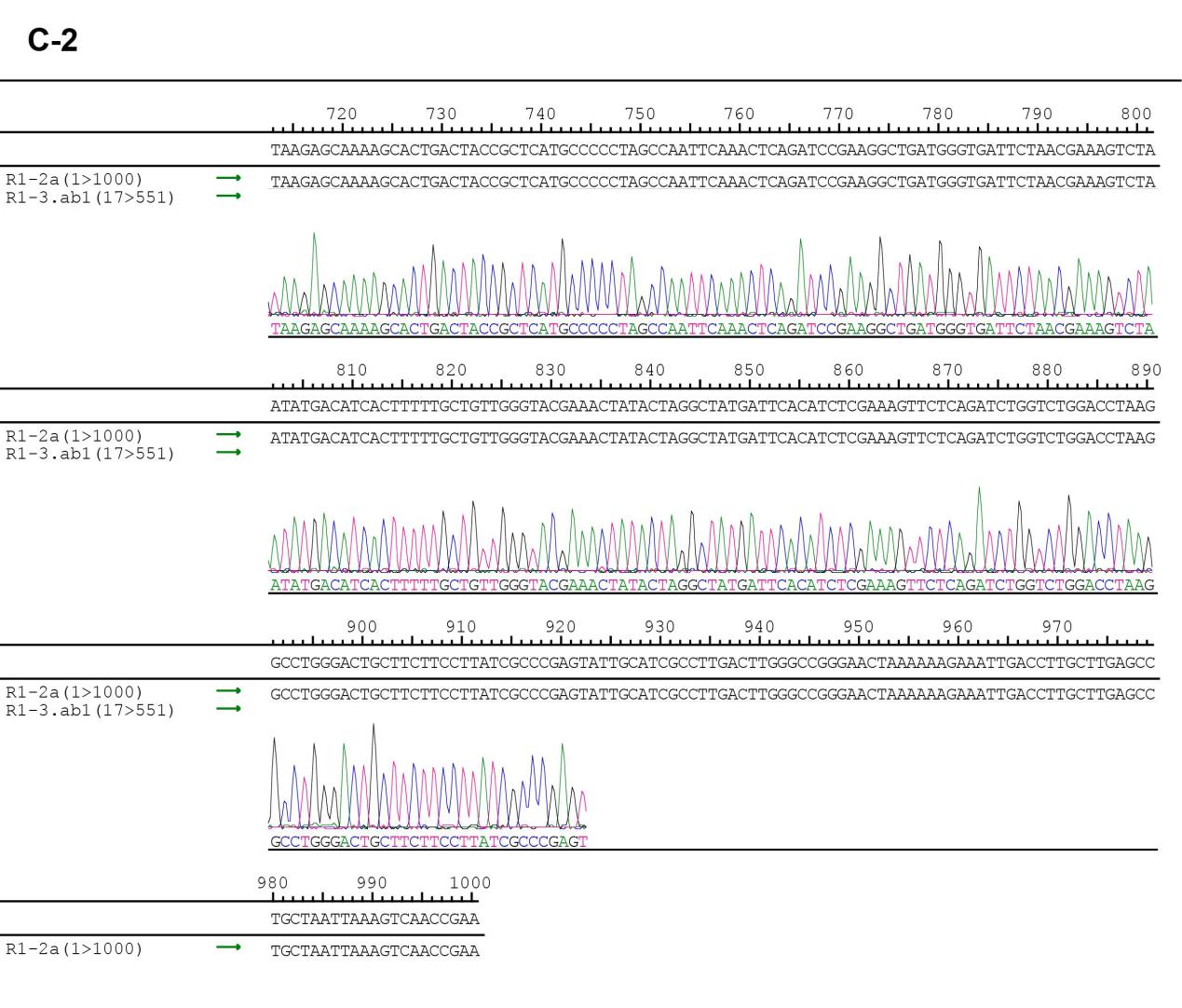


**Figure S4**. Boundary verification of repeated fragment (C: R1-3).


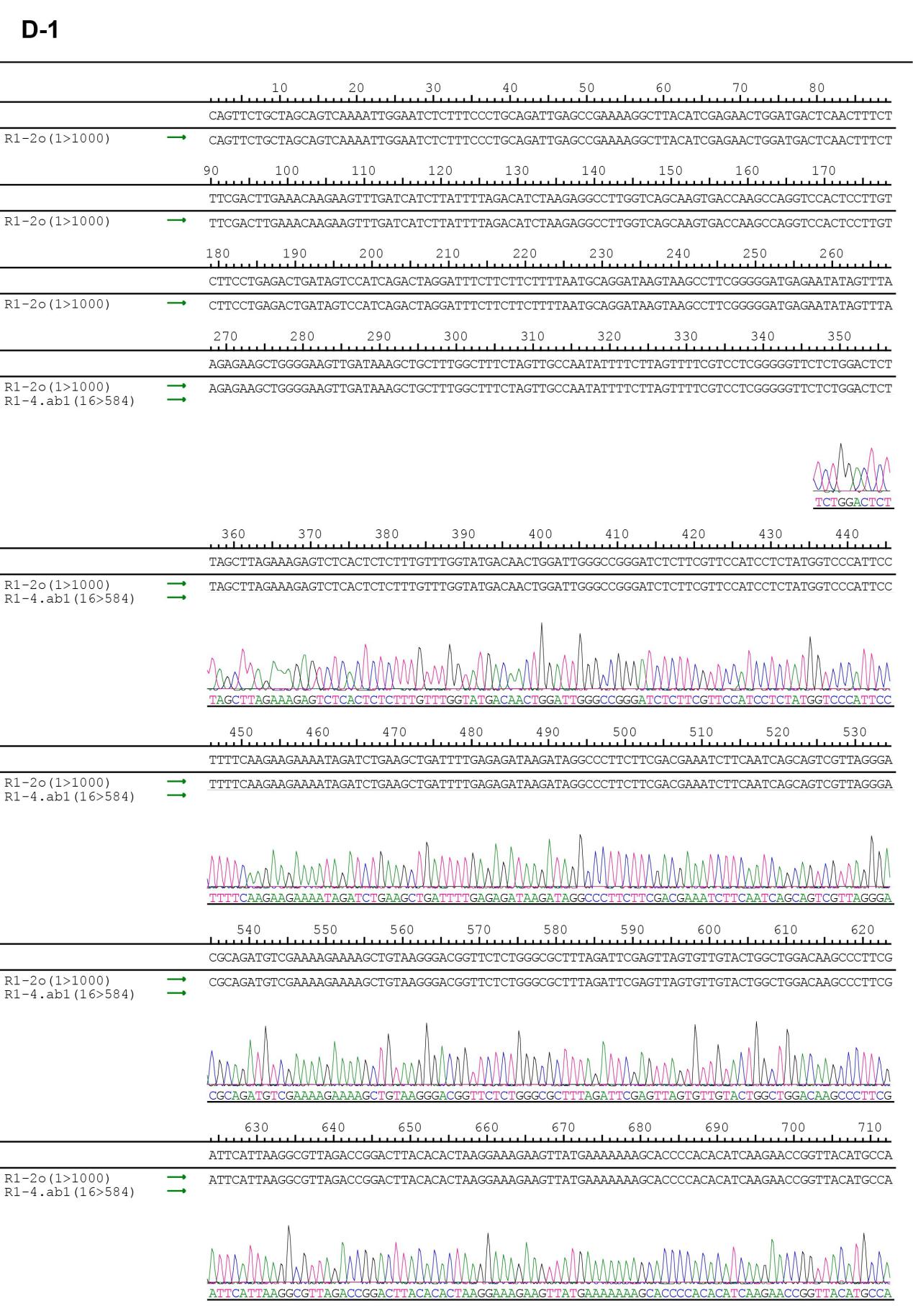

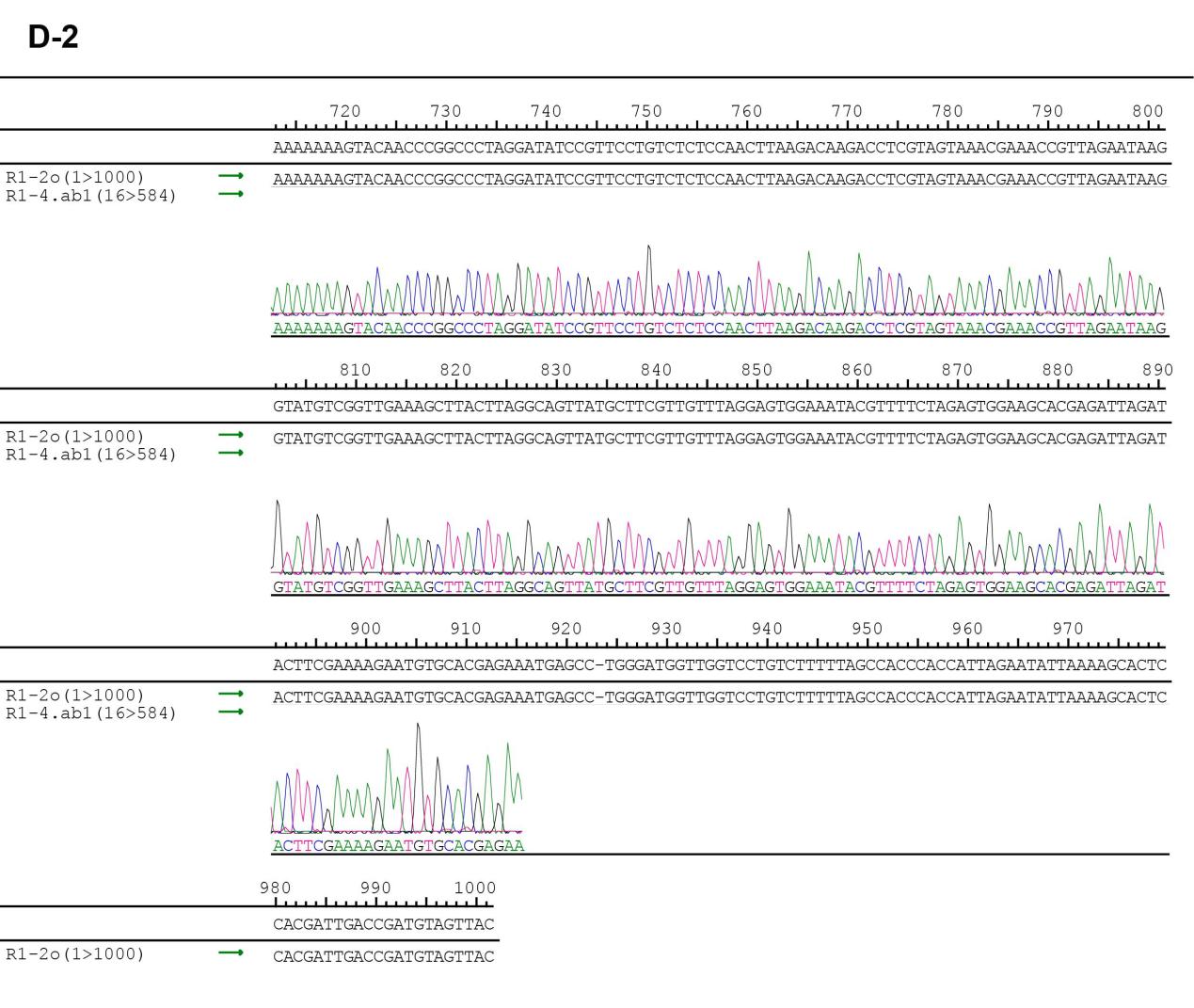


**Figure S4**. Boundary verification of repeated fragment (D: R1-4).


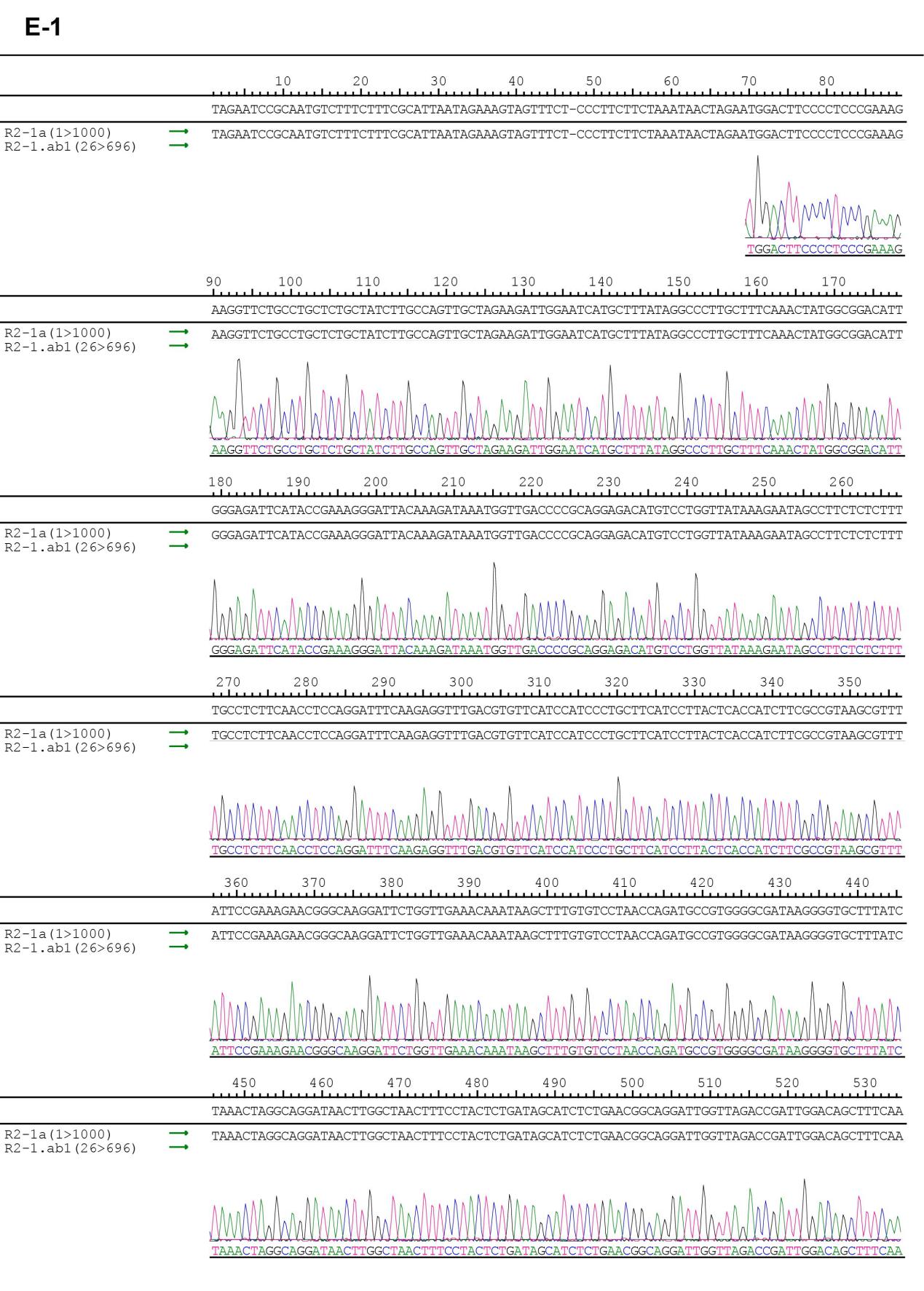

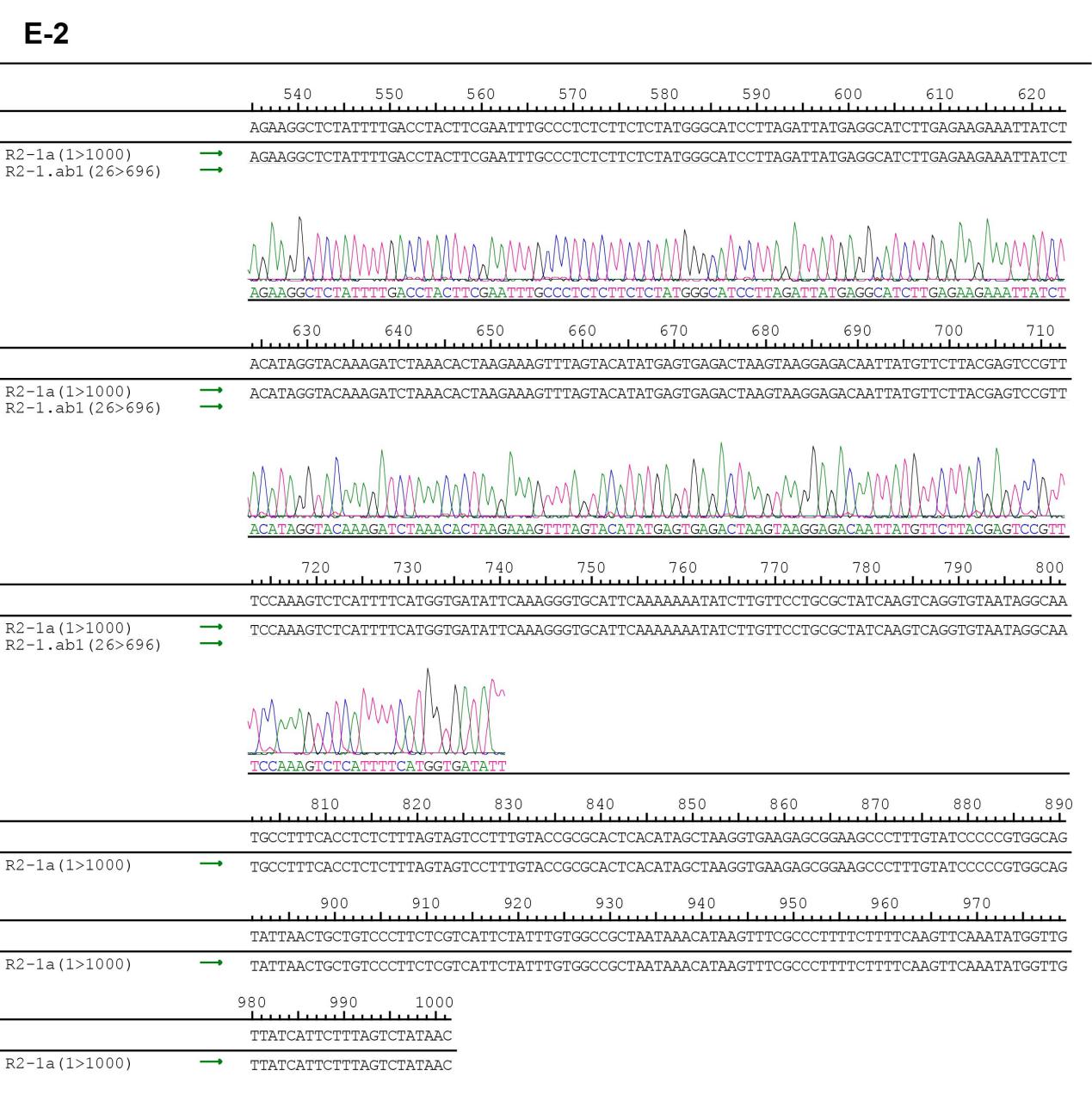


**Figure S4**. Boundary verification of repeated fragment (E: R2-1).


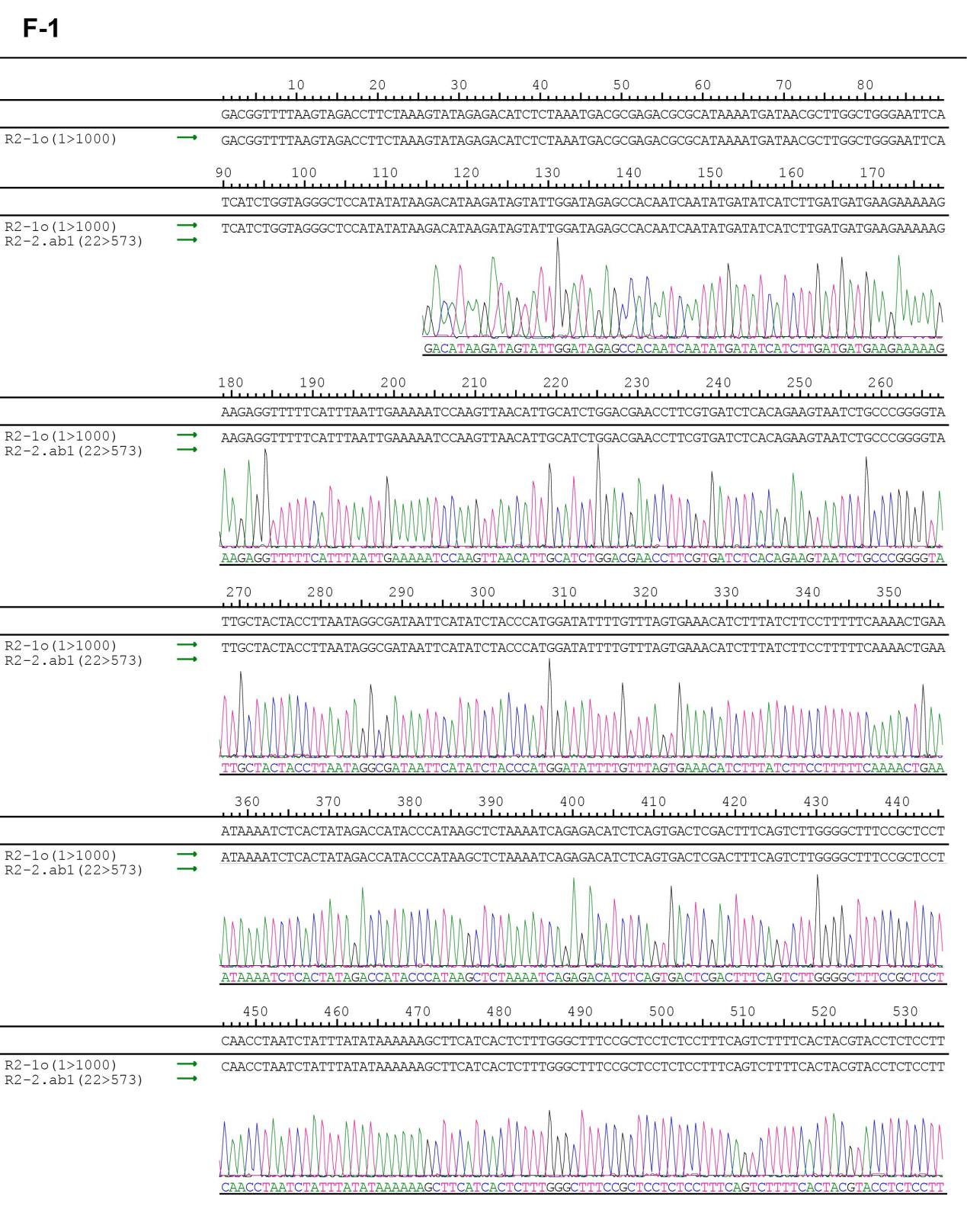

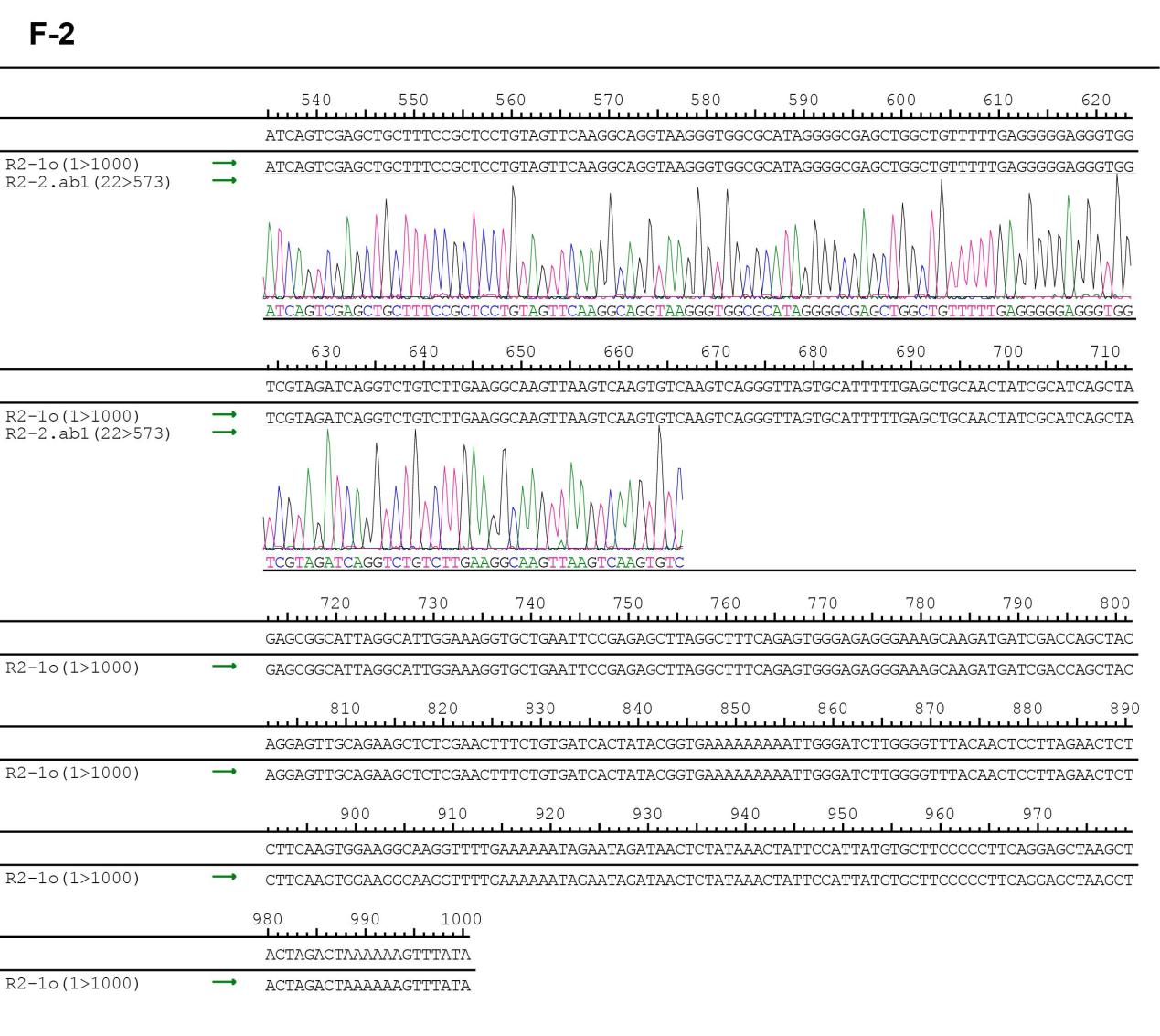


**Figure S4**. Boundary verification of repeated fragment (F: R2-2).


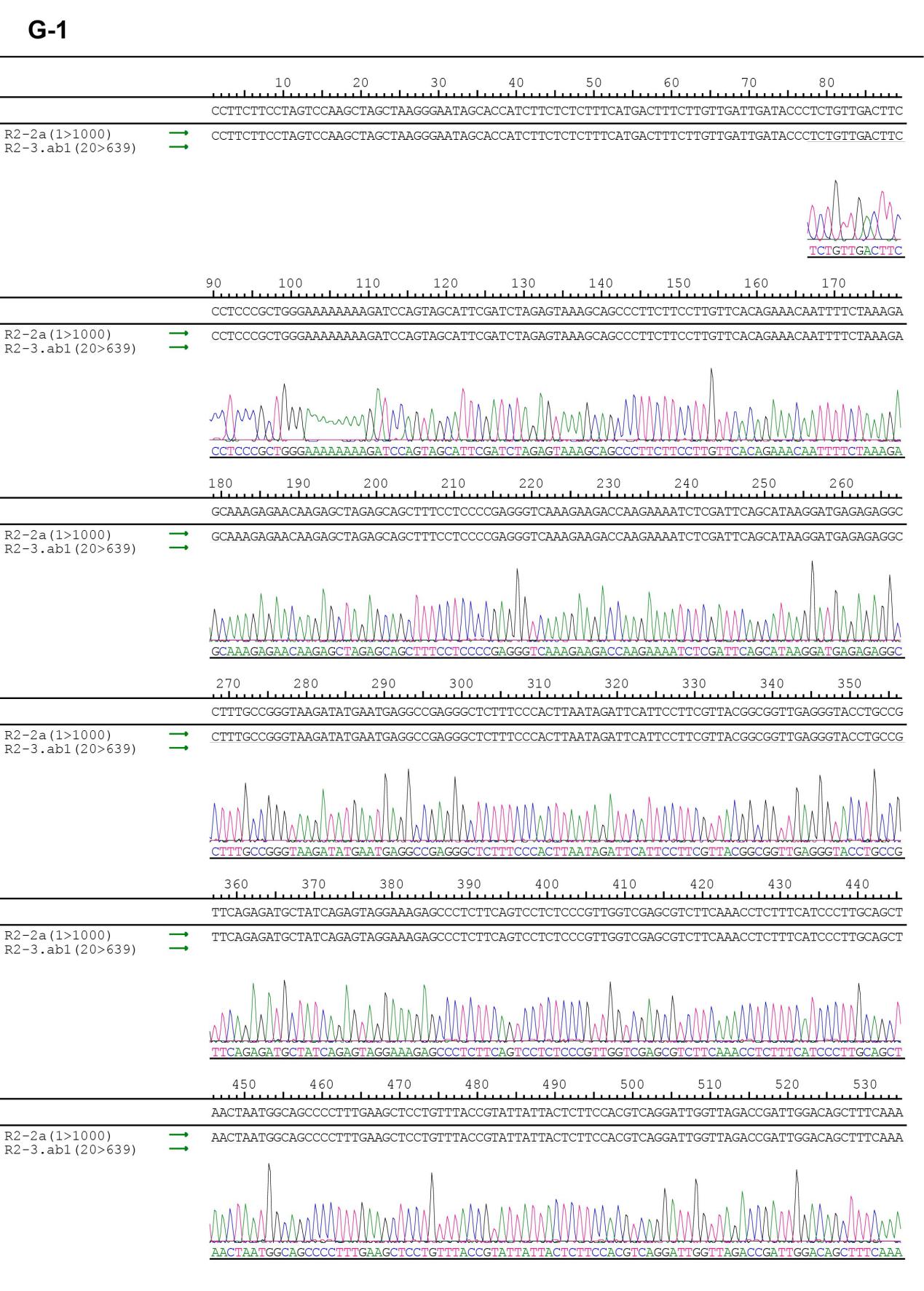

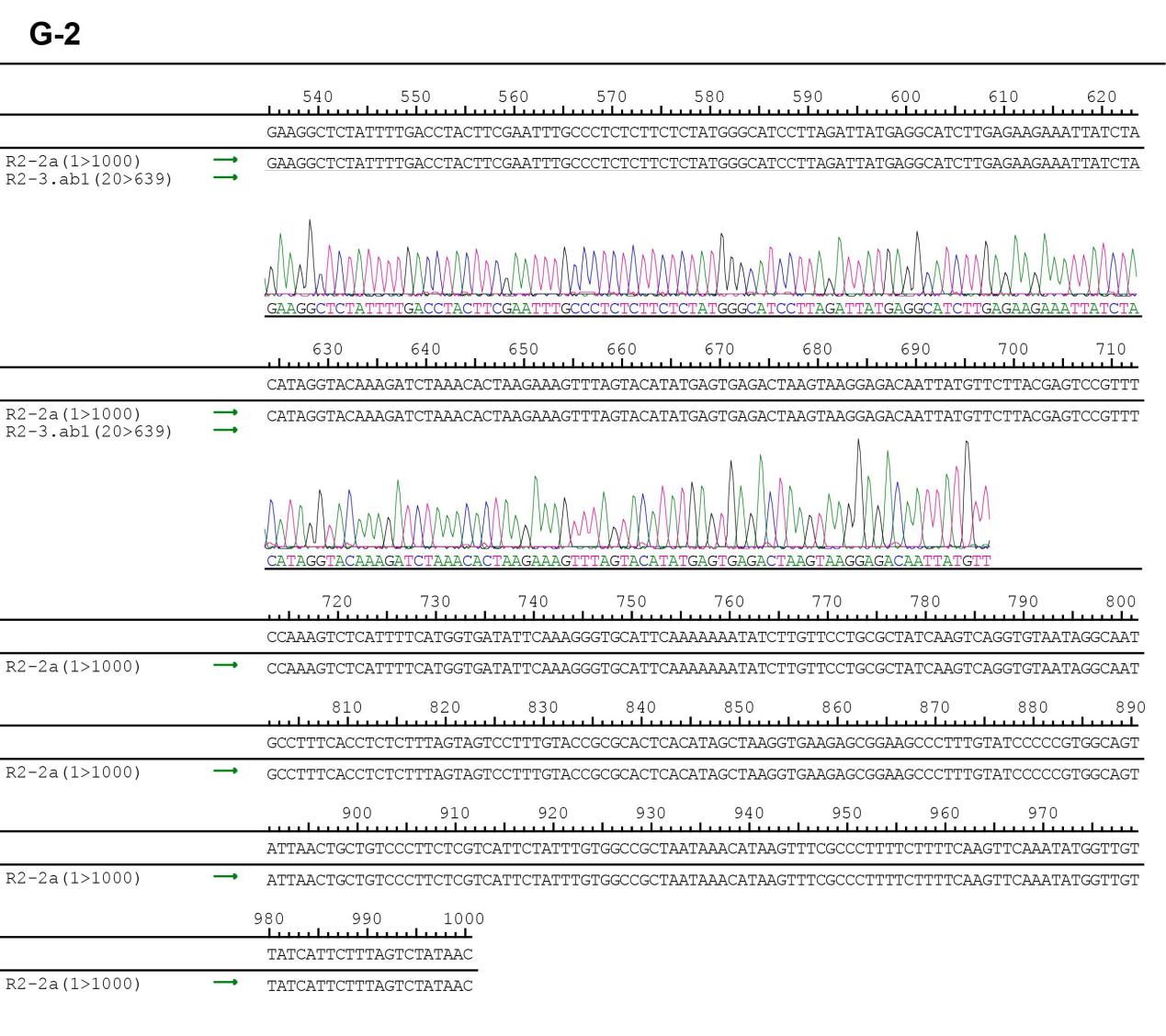


**Figure S4**. Boundary verification of repeated fragment (G: R2-3).


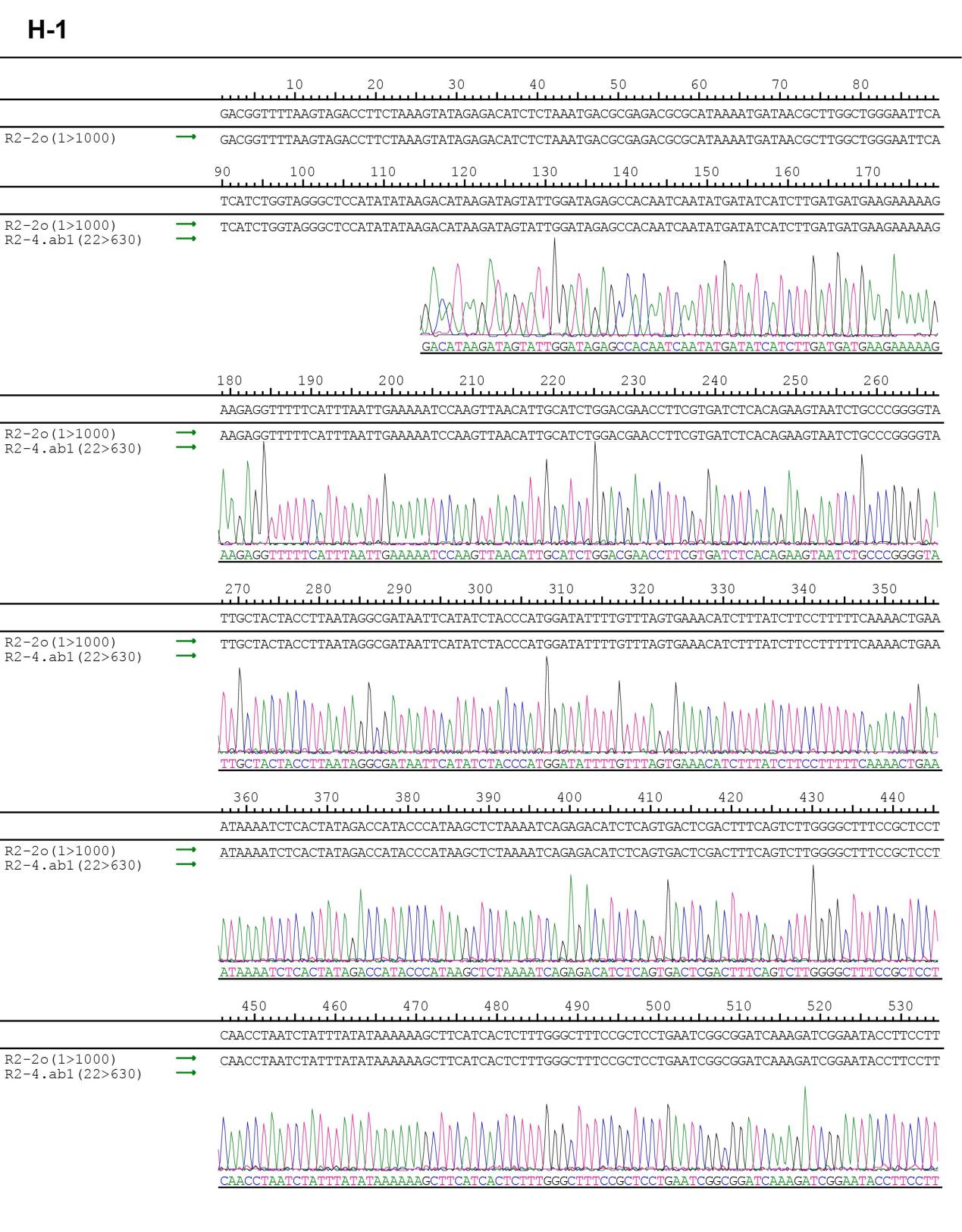

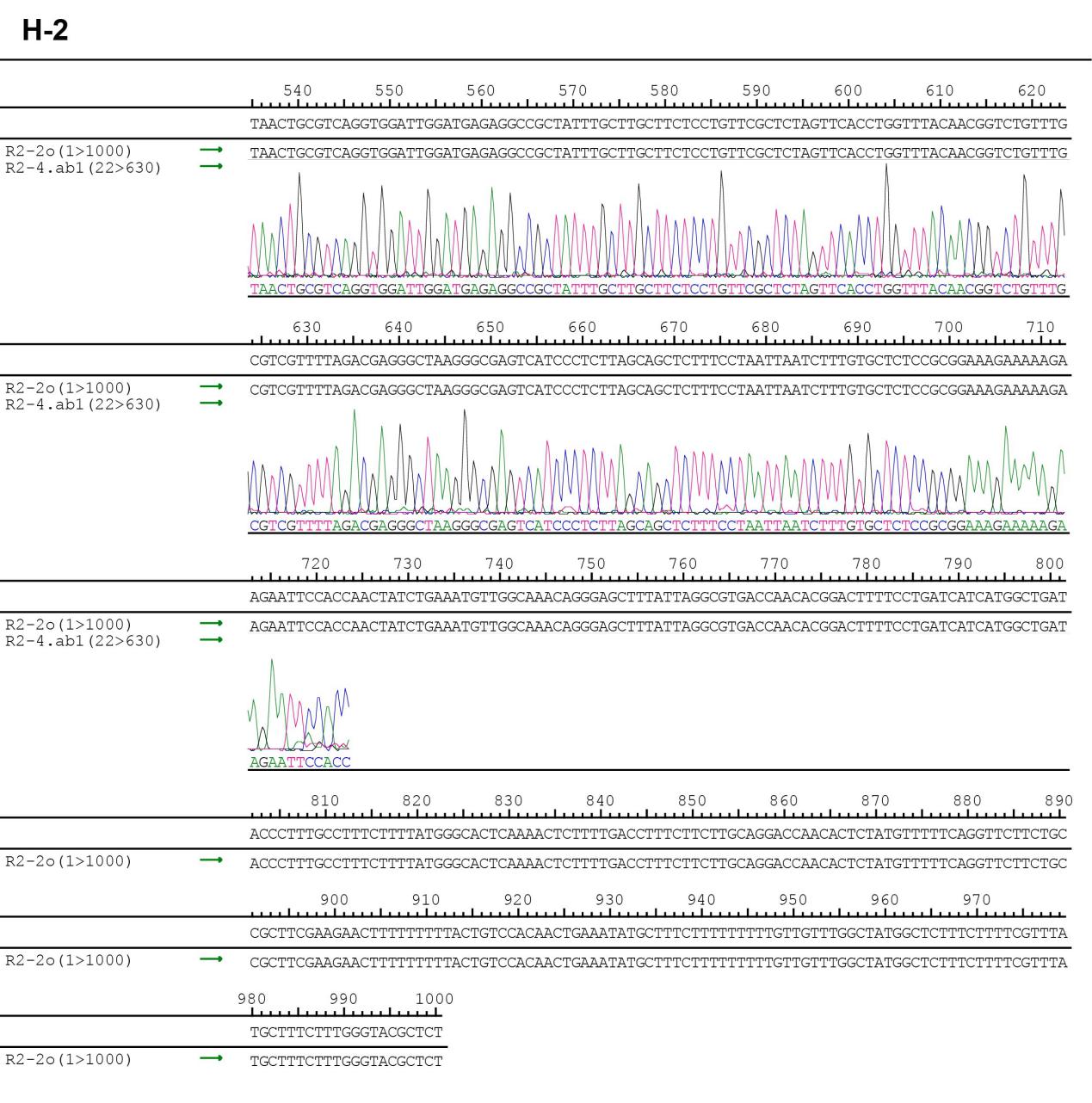


**Figure S4**. Boundary verification of repeated fragment (H: R2-4).


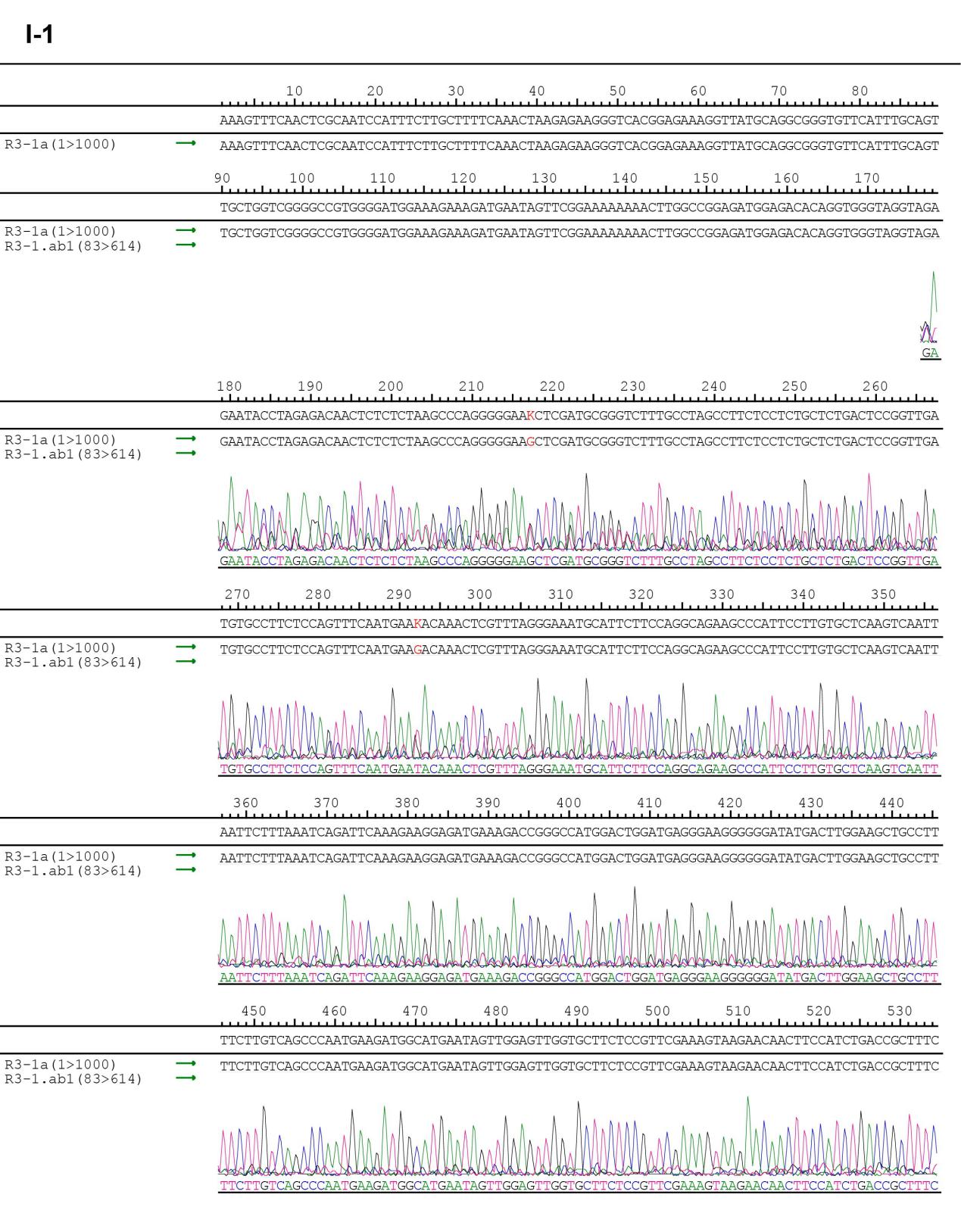

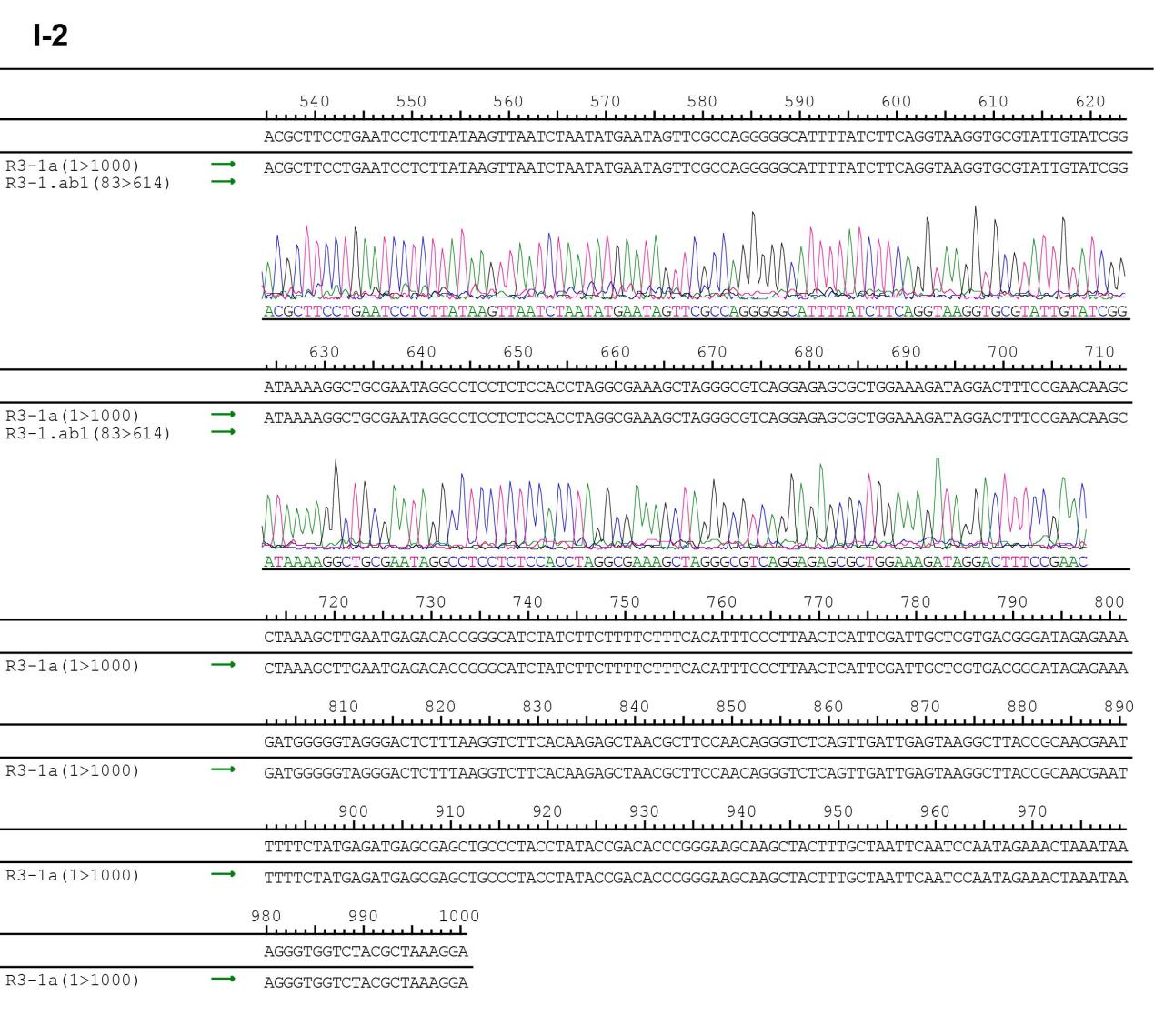


**Figure S4**. Boundary verification of repeated fragment (I: R3-1).


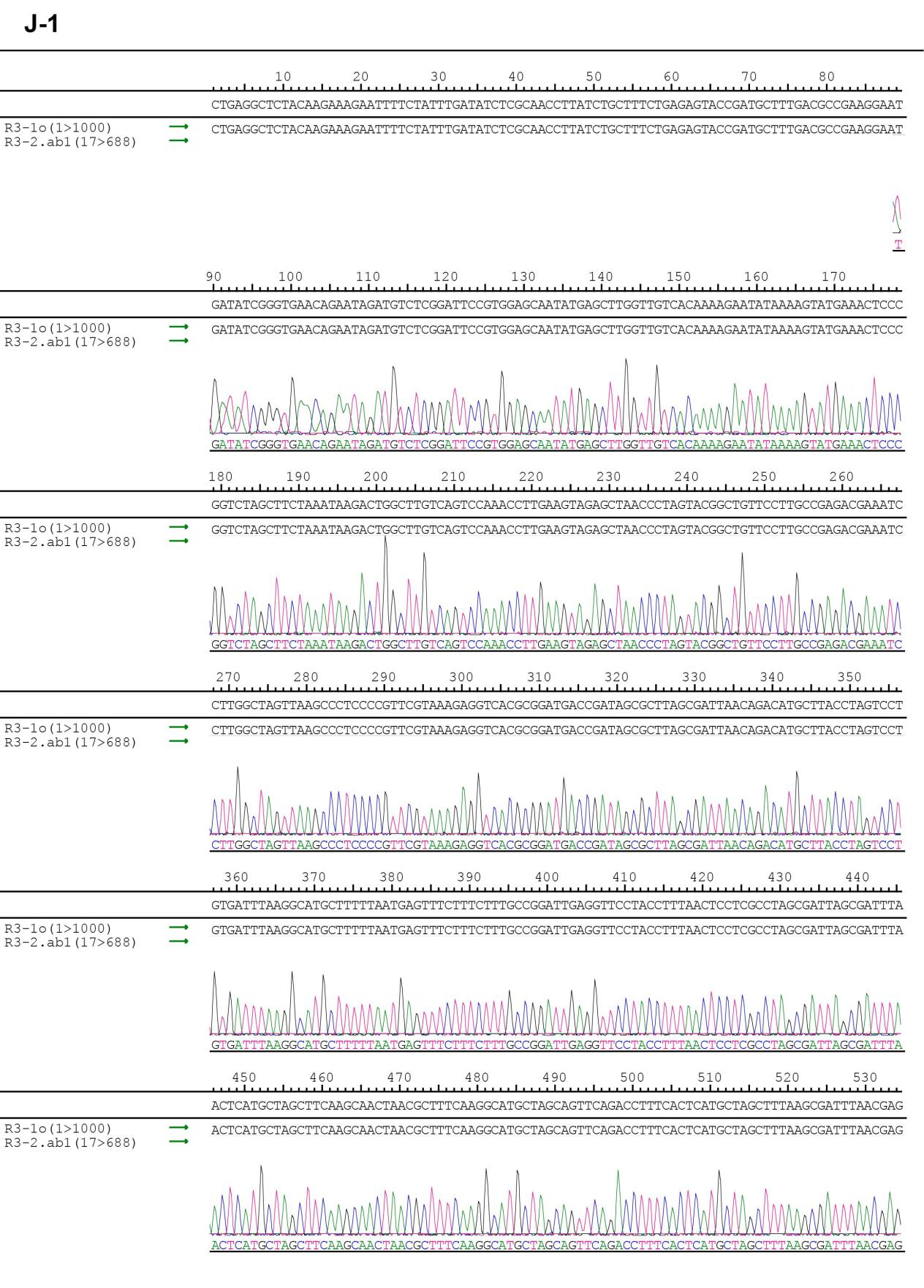

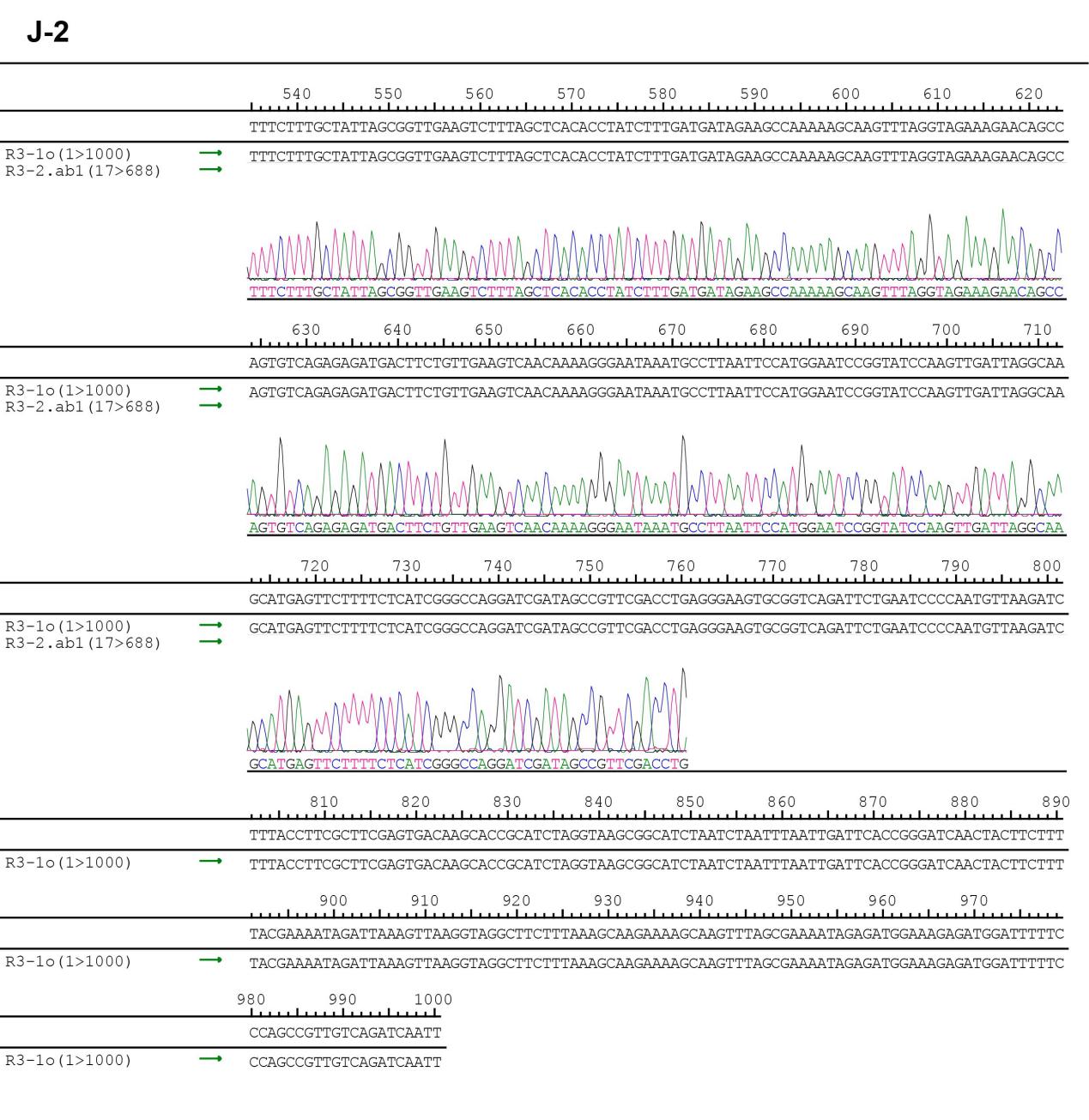


**Figure S4**. Boundary verification of repeated fragment (J: R3-2).


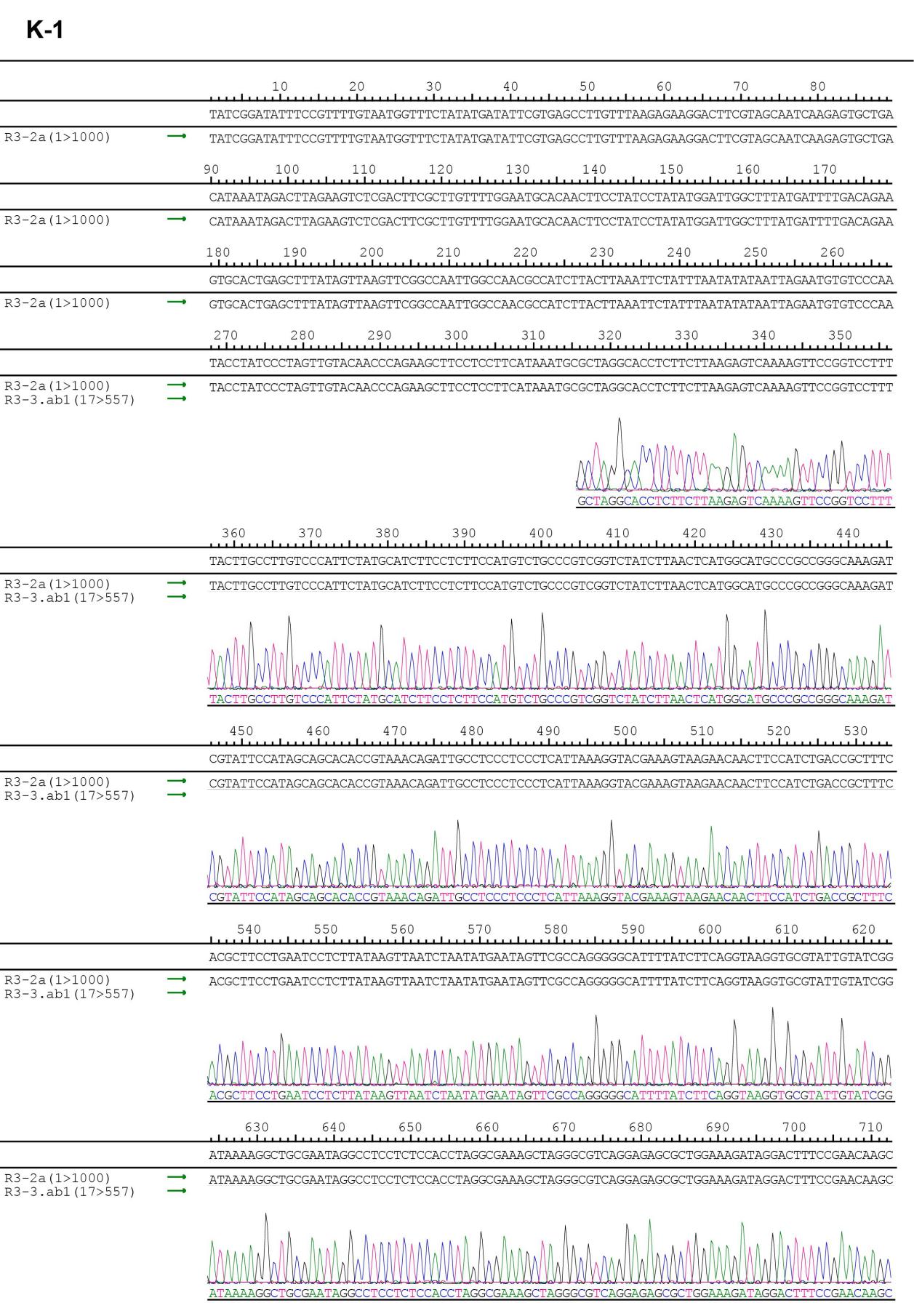

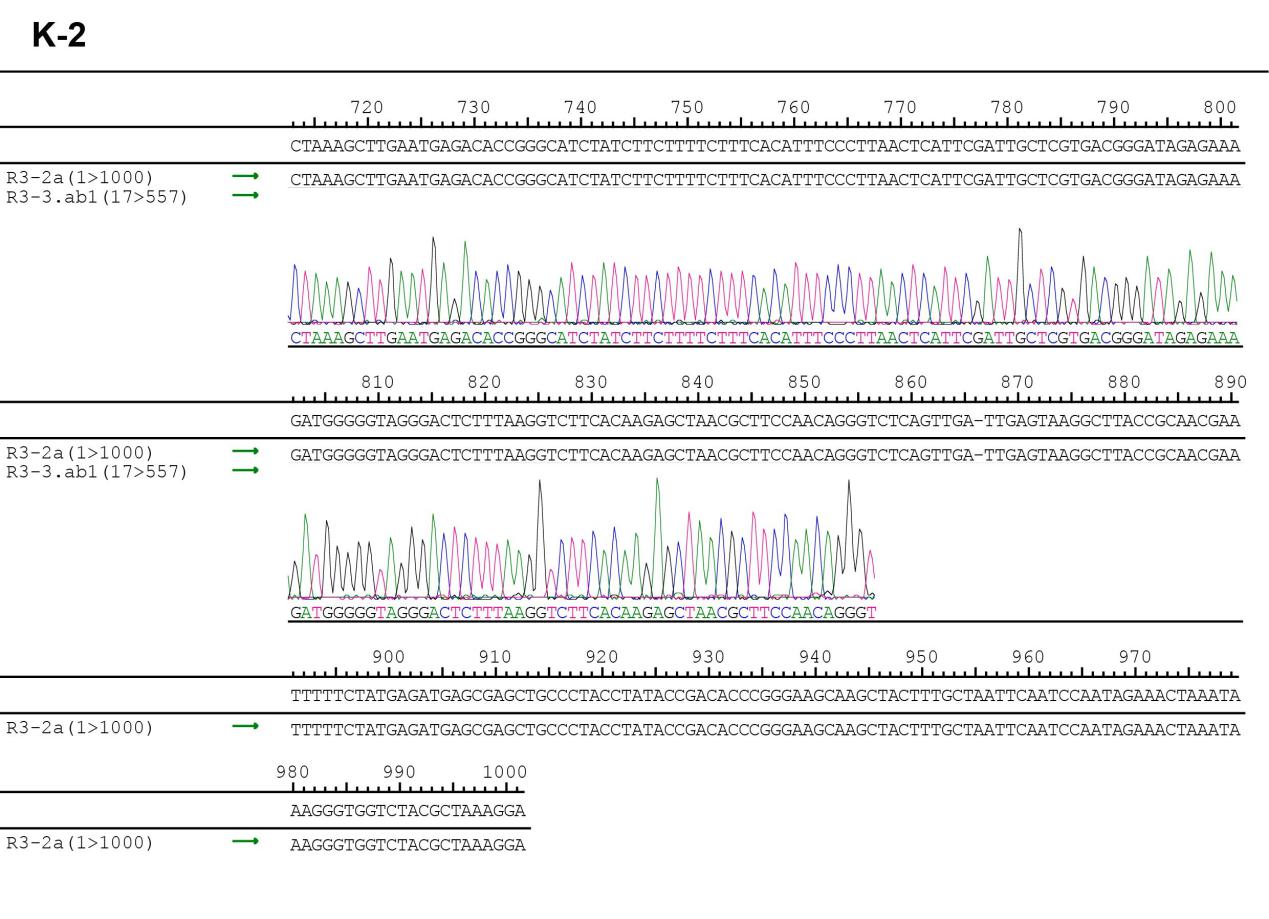


**Figure S4**. Boundary verification of repeated fragment (K: R3-3).


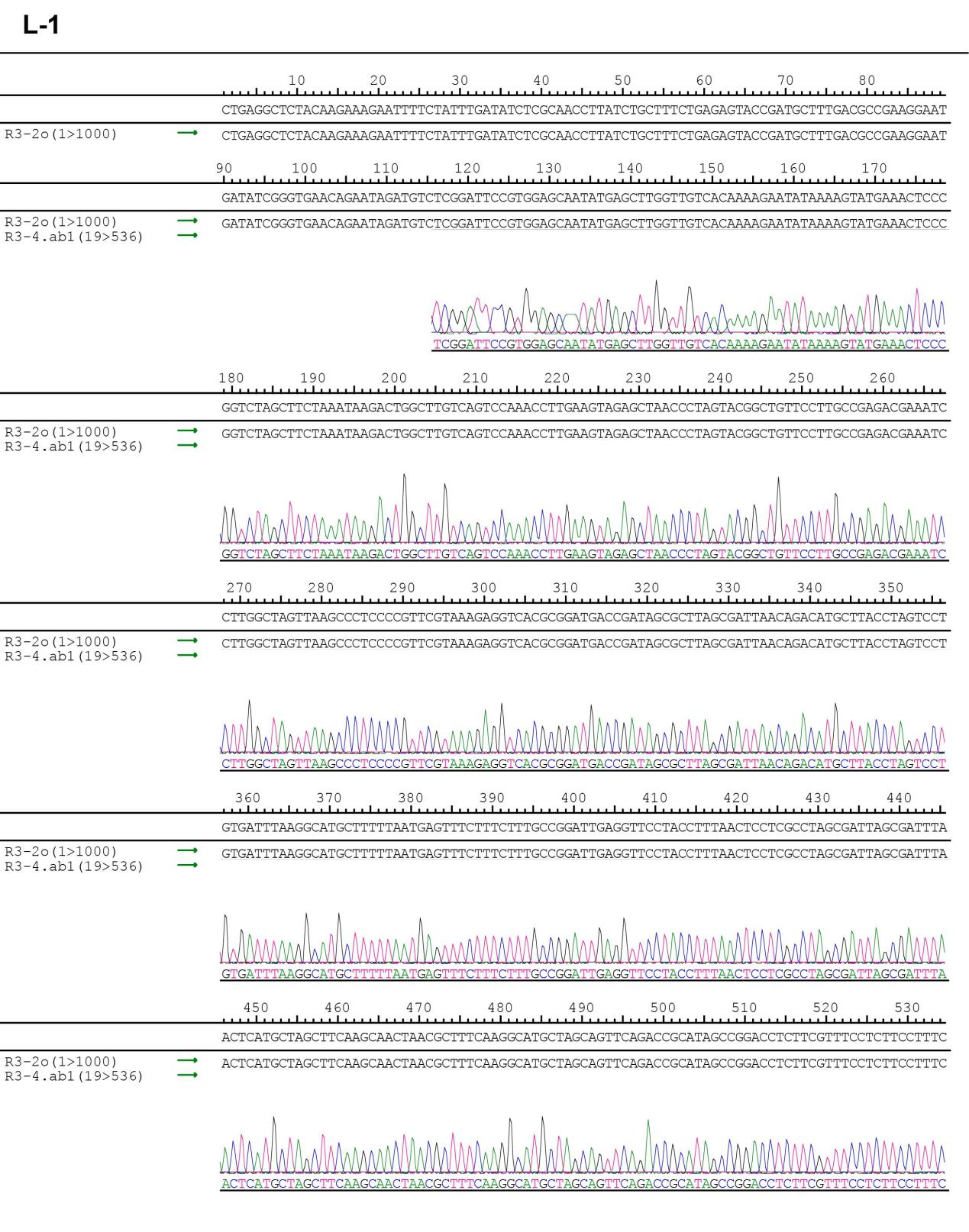

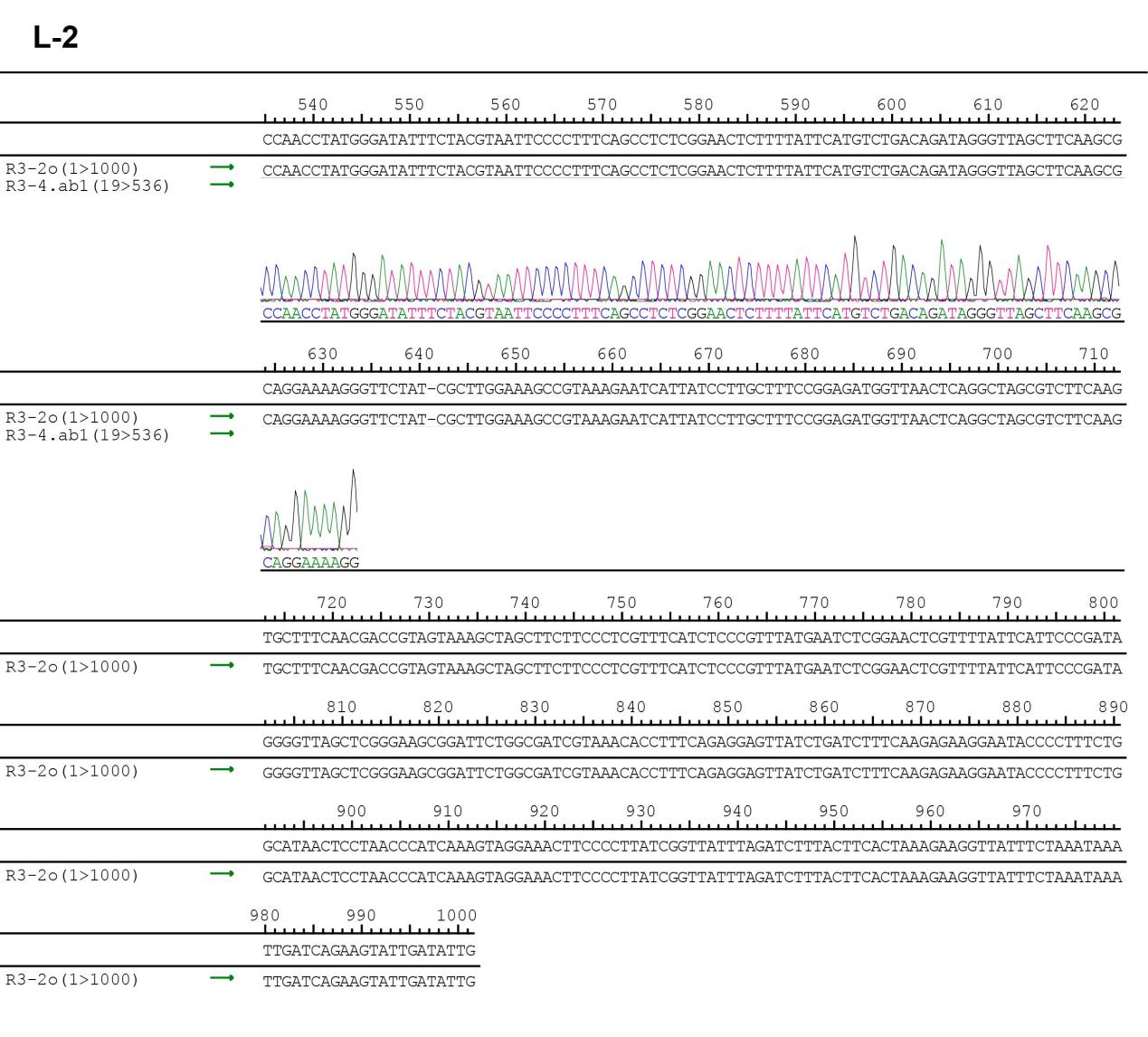


**Figure S4**. Boundary verification of repeated fragment (L: R3-4).
