# Supplementary material for: Complete mitogenome assembly of Selenicereus monacanthus revealed its molecular features, genome evolution, and phylogenetic implications
Source: BMC Plant Biol. 2023 Nov 4;23:541. doi: 10.1186/s12870-023-04529-9 (PMC10625231; doi:10.1186/s12870-023-04529-9)
Supplement: Supplementary file 7 — Supplementary Material 7 [file 12870_2023_4529_MOESM7_ESM.pdf]

Supplementary 1. gDNA and cDNA sequence comparison of editing site nad1-2

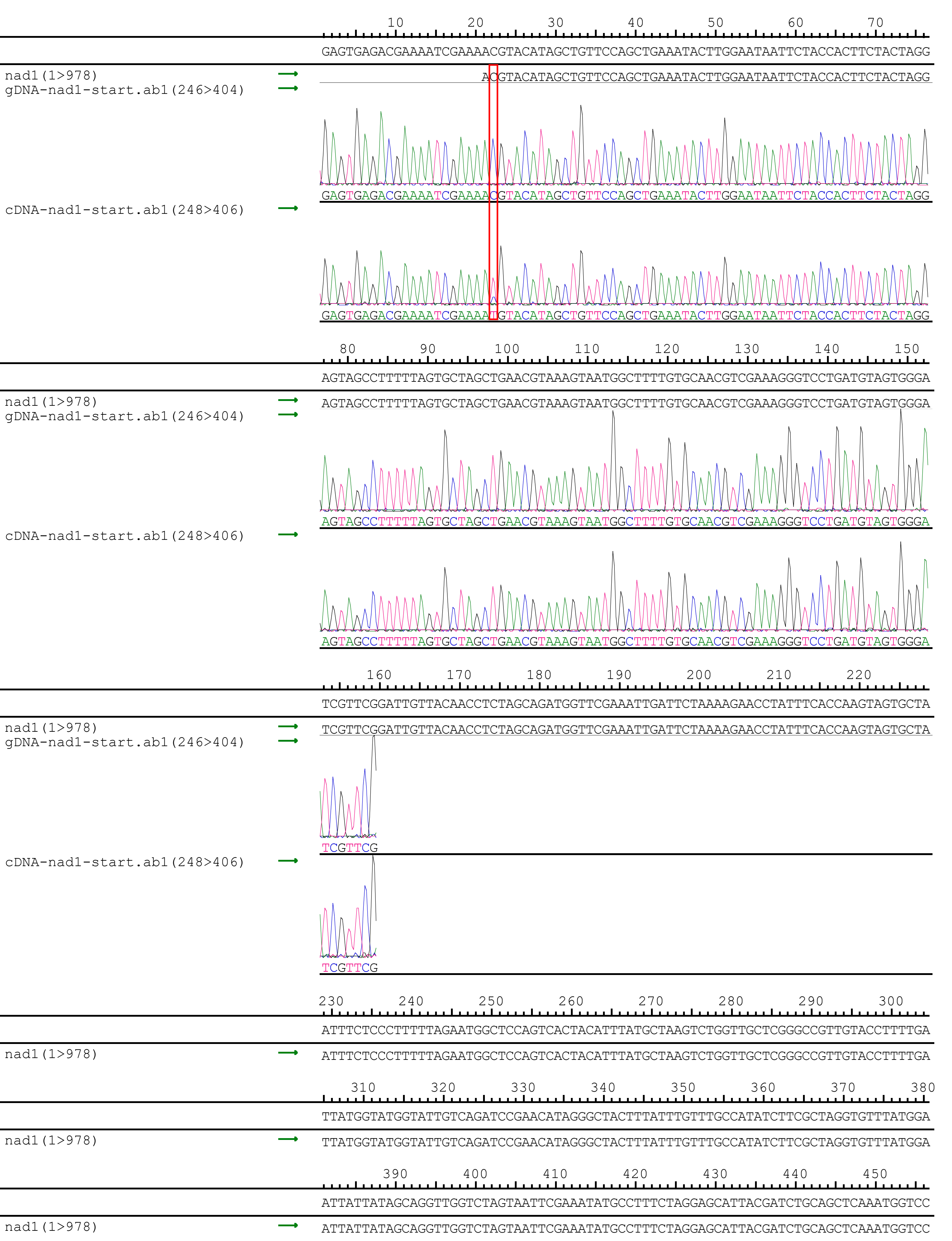

```
                        460       470       480       490       500       510       520       530
                        CTTATGAAGTCTCTATTGGTCTTATTCTTATTACTGTACTAATATGTGTAGGTCCTCGTAATTCGAGTGAGATTGT
nad1(1>978)         →   CTTATGAAGTCTCTATTGGTCTTATTCTTATTACTGTACTAATATGTGTAGGTCCTCGTAATTCGAGTGAGATTGT

                            540       550       560       570       580       590       600
                        CATGGCGCAAAAGCAGATATGGTCCGGTATTCCCTTGTTCCCTGTATTGGTTATGTTCTTTATTTCTTGTTTAGCA
nad1(1>978)         →   CATGGCGCAAAAGCAGATATGGTCCGGTATTCCCTTGTTCCCTGTATTGGTTATGTTCTTTATTTCTTGTTTAGCA

                        610       620       630       640       650       660       670       680
                        GAAACTAATCGAGCTCCGTTTGATCTCCCAGAAGCGGAAGCTGAATTAGTTGCAGGCTATAATGTAGAATATTCTT
nad1(1>978)         →   GAAACTAATCGAGCTCCGTTTGATCTCCCAGAAGCGGAAGCTGAATTAGTTGCAGGCTATAATGTAGAATATTCTT

                            690       700       710       720       730       740       750       760
                        CAATGGGGTCTGCTCTTTTTTTTTTAGGAGAGTATGCCAATATGATCTTAATGAGTGGTCTATGCACATTGCTTTC
nad1(1>978)         →   CAATGGGGTCTGCTCTTTTTTTTTTAGGAGAGTATGCCAATATGATCTTAATGAGTGGTCTATGCACATTGCTTTC

                                770       780       790       800       810       820       830
                        TCCAGGAGGTTGGCCGCCTATCCTAGATCTTCCCATTTCCAAAAGGATCCCGGGCTCAATCTGGTTTAGTATCAAG
nad1(1>978)         →   TCCAGGAGGTTGGCCGCCTATCCTAGATCTTCCCATTTCCAAAAGGATCCCGGGCTCAATCTGGTTTAGTATCAAG

                        840       850       860       870       880       890       900       910
                        GTGATTCTCTTTCTCTTTCTATATATATGGGTCCGTGCAGCATTTCCACGATATCGTTATGATCAATTAATGGGAC
nad1(1>978)         →   GTGATTCTCTTTCTCTTTCTATATATATGGGTCCGTGCAGCATTTCCACGATATCGTTATGATCAATTAATGGGAC

                            920       930       940       950       960       970       980
                        TTGGCCGGAAAGTGTTCTTGCCTCTATCATTAGCTCGGGTAGTCGCAGTTTCTGGTGTTTTAGTCACCTTTCAATG
nad1(1>978)         →   TTGGCCGGAAAGTGTTCTTGCCTCTATCATTAGCTCGGGTAGTCGCAGTTTCTGGTGTTTTAGTCACCTTTCAATG

                        990
                        GCTCCCTTAA
nad1(1>978)         →   GCTCCCTTAA
```
