# Supplementary material for: Complete mitogenome assembly of Selenicereus monacanthus revealed its molecular features, genome evolution, and phylogenetic implications
Source: BMC Plant Biol. 2023 Nov 4;23:541. doi: 10.1186/s12870-023-04529-9 (PMC10625231; doi:10.1186/s12870-023-04529-9)
Supplement: Supplementary file 10 — Supplementary Material 10 [file 12870_2023_4529_MOESM10_ESM.pdf]

Supplementary 4. gDNA and cDNA sequence comparison of editing site ccmFC-1309

```
                 10        20        30        40        50        60        70
                 ATGGTCCAACTACATAACTTTTTCTTTTTCATTACTTCCATGGTCGTGCCTTGTGGCACGGCAGCACCCGTACTATT
ccmFC(1>1311) →  ATGGTCCAACTACATAACTTTTTCTTTTTCATTACTTCCATGGTCGTGCCTTGTGGCACGGCAGCACCCGTACTATT

                 80        90        100       110       120       130       140       150
                 GAAATGGTTCGTCAGTAGAGATGTTCCCACAGGTGCCCCTTTTTCCAATGGTACTTTAATTCCTATTCTTATCCCTT
ccmFC(1>1311) →  GAAATGGTTCGTCAGTAGAGATGTTCCCACAGGTGCCCCTTTTTCCAATGGTACTTTAATTCCTATTCTTATCCCTT

                 160       170       180       190       200       210       220       230
                 CATTCCTTCTTTTGGTCTATCTACATTCCAGGAAATTCATACGCTCCATGGACGGAGTCAAAAGTGGAGTCTTGGTC
ccmFC(1>1311) →  CATTCCTTCTTTTGGTCTATCTACATTCCAGGAAATTCATACGCTCCATGGACGGAGTCAAAAGTGGAGTCTTGGTC

                 240       250       260       270       280       290       300
                 AGAGCAAGCTGCCCTATTTTATTACCAGACATAATTGGGAGAAGCTCATCCGAAACGTTAGCGTTAAAGGCCTTATT
ccmFC(1>1311) →  AGAGCAAGCTGCCCTATTTTATTACCAGACATAATTGGGAGAAGCTCATCCGAAACGTTAGCGTTAAAGGCCTTATT

                 310       320       330       340       350       360       370       380
                 TTGTTTCGTTCCCATTCTTCATTTTCTTCTTCTCGAATCCAAGGGGGACTTCCCATATTTAGAATCTTTTTGCGGTG
ccmFC(1>1311) →  TTGTTTCGTTCCCATTCTTCATTTTCTTCTTCTCGAATCCAAGGGGGACTTCCCATATTTAGAATCTTTTTGCGGTG

                 390       400       410       420       430       440       450       460
                 TGCTCCGTTTACTATTCTTTCGTACTTTCTTCTCTTTACCACGCGATAGGTCAGCGAAGCGTGAGCGGGCGCGGAGA
ccmFC(1>1311) →  TGCTCCGTTTACTATTCTTTCGTACTTTCTTCTCTTTACCACGCGATAGGTCAGCGAAGCGTGAGCGGGCGCGGAGA

                 470       480       490       500       510       520       530
                 AGAAAAGGCCAAACCCAAAGGCCTAACGGGAATGAGCAACGACGAAATGACTTCATAAAGTGCCCCGGGCGCCCCCA
ccmFC(1>1311) →  AGAAAAGGCCAAACCCAAAGGCCTAACGGGAATGAGCAACGACGAAATGACTTCATAAAGTGCCCCGGGCGCCCCCA

                 540       550       560       570       580       590       600       610
                 TGACGAAAGAAGGGTCGAAGGGTTTGGGCCTGTAGCTTTCCCCGTCCCCCCTTCGTCGGGTGGTGCTTGTGTGTGGG
ccmFC(1>1311) →  TGACGAAAGAAGGGTCGAAGGGTTTGGGCCTGTAGCTTTCCCCGTCCCCCCTTCGTCGGGTGGTGCTTGTGTGTGGG

                 620       630       640       650       660       670       680       690
                 GTGTGCCACCTGAAATCGGGCTTGAAGCTCCCGCCTTACCAACGAGCCGACAGCTGATGGCTGTTGGTCACGACTAC
ccmFC(1>1311) →  GTGTGCCACCTGAAATCGGGCTTGAAGCTCCCGCCTTACCAACGAGCCGACAGCTGATGGCTGTTGGTCACGACTAC

                 700       710       720       730       740       750       760       770
                 TACCAAAAAGTGAACATGAAGATGAATATTTCACATGGAGGAGTGTGCATCTTTATGTTGGGTGTTCTTCTGTCTAA
ccmFC(1>1311) →  TACCAAAAAGTGAACATGAAGATGAATATTTCACATGGAGGAGTGTGCATCTTTATGTTGGGTGTTCTTCTGTCTAA

                 780       790       800       810       820       830       840
                 CACAAAGAAGATACAGTTCACTCAACGATTGCCTTTGGGTTCCGAACTCCATATGGGGAAGGAACGTTGTTGTTTGC
ccmFC(1>1311) →  CACAAAGAAGATACAGTTCACTCAACGATTGCCTTTGGGTTCCGAACTCCATATGGGGAAGGAACGTTGTTGTTTGC

                 850       860       870       880       890       900       910       920
                 GGGGTCTCGATCATTTACATGGACCCACTTTTCATGGAATTTGTGGGAATTTGATGATCTATAAACCGTCCTTAACG
ccmFC(1>1311) →  GGGGTCTCGATCATTTACATGGACCCACTTTTCATGGAATTTGTGGGAATTTGATGATCTATAAACCGTCCTTAACG

                 930       940       950       960       970       980       990       1000
                 AACGATCGGCTCATCTTTGAGCATGATGAATCACTTCGTGCCGACCTGTTGTCAATAAACTTTTTGGCCTCATATGA
ccmFC(1>1311) →  AACGATCGGCTCATCTTTGAGCATGATGAATCACTTCGTGCCGACCTGTTGTCAATAAACTTTTTGGCCTCATATGA

                 1010      1020      1030      1040      1050      1060      1070
                 GAATGGAAAACTGGAGCTTCATCGGTGGATGAAGAATCGCGAACATAATAATTTATGGTTAAGCATGTTCCCAGAAA
ccmFC(1>1311) →  GAATGGAAAACTGGAGCTTCATCGGTGGATGAAGAATCGCGAACATAATAATTTATGGTTAAGCATGTTCCCAGAAA
```

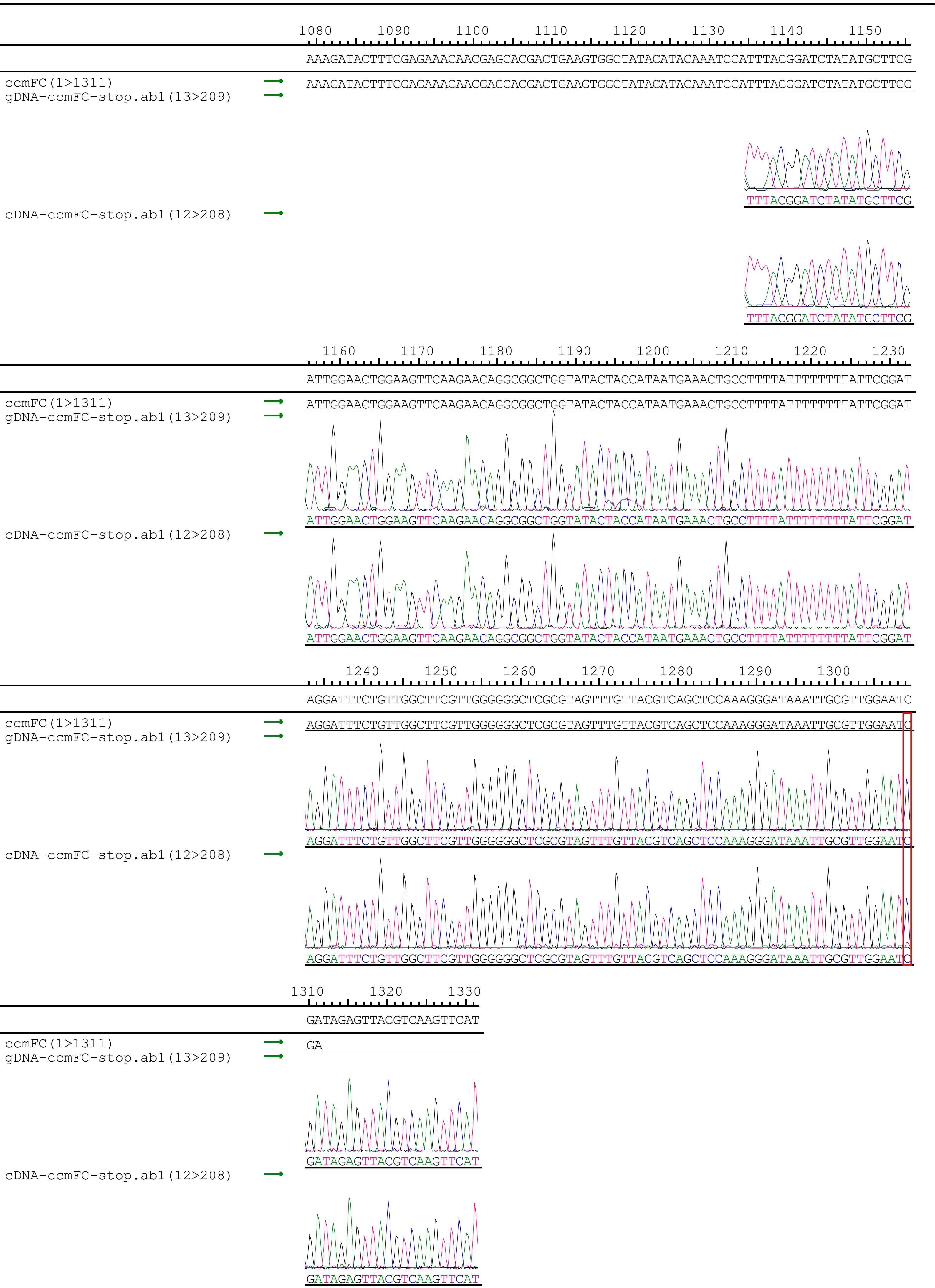
1080 1090 1100 1110 1120 1130 1140 1150
AAAGATACTTTCGAGAAACAACGAGCACGACTGAAGTGGCTATACATACAAATCCATTTACGGATCTATATGCTTCG
ccmFC(1>1311)
AAAGATACTTTCGAGAAACAACGAGCACGACTGAAGTGGCTATACATACAAATCCATTTACGGATCTATATGCTTCG
gDNA-ccmFC-stop.ab1(13>209)
TTTACGGATCTATATGCTTCG
cDNA-ccmFC-stop.ab1(12>208)
TTTACGGATCTATATGCTTCG
1160 1170 1180 1190 1200 1210 1220 1230
ATTGGAACTGGAAGTTCAAGAACAGGCGGCTGGTATACTACCATAATGAAACTGCCTTTTATTTTTTTTATTCGGAT
ccmFC(1>1311)
ATTGGAACTGGAAGTTCAAGAACAGGCGGCTGGTATACTACCATAATGAAACTGCCTTTTATTTTTTTTATTCGGAT
gDNA-ccmFC-stop.ab1(13>209)
ATTGGAACTGGAAGTTCAAGAACAGGCGGCTGGTATACTACCATAATGAAACTGCCTTTTATTTTTTTTATTCGGAT
cDNA-ccmFC-stop.ab1(12>208)
ATTGGAACTGGAAGTTCAAGAACAGGCGGCTGGTATACTACCATAATGAAACTGCCTTTTATTTTTTTTATTCGGAT
1240 1250 1260 1270 1280 1290 1300
AGGATTTCTGTTGGCTTCGTTGGGGGGCTCGCGTAGTTTGTTACGTCAGCTCCAAAGGGATAAATTGCGTTGGAATC
ccmFC(1>1311)
AGGATTTCTGTTGGCTTCGTTGGGGGGCTCGCGTAGTTTGTTACGTCAGCTCCAAAGGGATAAATTGCGTTGGAATC
gDNA-ccmFC-stop.ab1(13>209)
AGGATTTCTGTTGGCTTCGTTGGGGGGCTCGCGTAGTTTGTTACGTCAGCTCCAAAGGGATAAATTGCGTTGGAATC
cDNA-ccmFC-stop.ab1(12>208)
AGGATTTCTGTTGGCTTCGTTGGGGGGCTCGCGTAGTTTGTTACGTCAGCTCCAAAGGGATAAATTGCGTTGGAATC
1310 1320 1330
GATAGAGTTACGTCAAGTTCAT
ccmFC(1>1311)
GA
gDNA-ccmFC-stop.ab1(13>209)
GATAGAGTTACGTCAAGTTCAT
cDNA-ccmFC-stop.ab1(12>208)
GATAGAGTTACGTCAAGTTCAT
