# Supplementary material for: Complete mitogenome assembly of Selenicereus monacanthus revealed its molecular features, genome evolution, and phylogenetic implications
Source: BMC Plant Biol. 2023 Nov 4;23:541. doi: 10.1186/s12870-023-04529-9 (PMC10625231; doi:10.1186/s12870-023-04529-9)
Supplement: Supplementary file 11 — Supplementary Material 11 [file 12870_2023_4529_MOESM11_ESM.docx]

**Supplementary Information**

The online version contains supplementary material available at https://doi.org/

**Figure S1.** Codon usage preference of protein-coding genes in the *Selenicereus monacanthus*. **Figure S2.** Repeats analysis of the mitochondrial genome in the *Selenicereus monacanthus*. **Figure S3.** The recombination structure prediction (**A**) and verification (**B**) in *Selenicereus monacanthus*. **Figure S4.** Boundary verification of repeated fragment. **Table S1.** Relative synonymous codon usage (RSCU) of each amino acid pair in the mitochondrial genome of *Selenicereus monacanthus*. **Table S2.** SSRs in the mitochondrial genome of *Selenicereus monacanthus*. **Table S3.** Tandem repeat sequences in the mitochondrial genome of *Selenicereus monacanthus*. **Table S4.** Dispersed repeat sequences in the mitochondrial genome of *Selenicereus monacanthus*. **Table S5.** RNA editing events predicted in *Selenicereus monacanthus* mitochondrial genome. **Table S6.** Fragments transferred from chloroplast to mitochondria in the *Selenicereus monacanthus*. **Table S7.** Sequence collinearity of the mitochondrial genome of *Selenicereus monacanthus* and related genera. **Table S8.** RValidation primers for repeat‑mediated recombination sequence. **Table S9.** Primer sequences used for RNA editing. **Table S10.** Related genera of the *Selenicereus monacanthus* and their complete mitochondrial genome in GenBank.
